# A Detailed Thermodynamic Description of Ion Pair Binding by a Calix[4]arene Derivative Containing Urea and Amide Functionalities

**DOI:** 10.3390/molecules30112464

**Published:** 2025-06-04

**Authors:** Marija Cvetnić, Tamara Rinkovec, Robert Vianello, Gordan Horvat, Nikola Bregović, Vladislav Tomišić

**Affiliations:** 1 Division of Physical Chemistry, Department of Chemistry, Faculty of Science, University of Zagreb, Horvatovac 102a, 10000 Zagreb, Croatia; marija.cvetnic@chem.pmf.hr (M.C.); ghorvat@chem.pmf.hr (G.H.); 2 Laboratory for the Computational Design and Synthesis of Functional Materials, Division of Organic Chemistry and Biochemistry, Ruđer Bošković Institute, Bijenička cesta 54, 10000 Zagreb, Croatia; tamara.rinkovec@irb.hr (T.R.); robert.vianello@irb.hr (R.V.)

**Keywords:** ion pair, calix[4]arene, cooperativity, binding thermodynamics, molecular dynamics, solution equilibria, acetonitrile, solubility

## Abstract

Receptors capable of binding both positive and negative ions are an important domain of supramolecular chemistry with valuable application potential. A Complete thermodynamic description of the equilibria related to ion pair recognition is beneficial in developing the optimized receptor systems, although it represents a difficult task that is rarely resolved due to various coupled processes. Here, we present a comprehensive study of ion pair (NaCl, NaHSO_4_, and NaH_2_PO_4_) binding by a ureido–amide calix[4]arene host in acetonitrile using a series of experimental techniques and molecular dynamics simulations. We devoted particular attention to characterizing the side processes (ion association and salt precipitation) and included them in the models describing ion pair complex formation. For this purpose, a multimethod approach (potentiometry, conductometry, ITC, flame AES) was employed, generating reliable data which provided insight into the thermodynamic effect of each included equilibrium. Positive cooperativity was observed in the context of NaCl and NaHSO_4_ binding by the studied calixarene. Computational results related to the NaCl complex in acetonitrile revealed that favorable Coulombic interactions, changes in affinity for solvent molecule inclusion, and intramolecular hydrogen bonding contributed to cation-induced cooperativity.

## 1. Introduction

Ion pair receptors hold immense potential across a wide range of applications, including salt extraction, salt solubilization, sensing (colorimetric, fluorometric, and electrochemical), transmembrane transport (via liposomes, bulk liquids, and supported liquids), molecular machines, switchable devices, logic gates, and self-assembly templates [1,2,3,4,5,6]. This versatility makes their exploration a particularly exciting area in supramolecular chemistry research. Ion pair receptors range from simple structured molecules to larger scaffolds and include crown ethers, calixpyrroles, and calixarenes, and more recently mechanically interlocked molecules (rotaxanes and catenanes), which benefit from the mechanical bond effect [7].

The receptors interact with ion pairs in different ways, depending on the type of the bound ion pair (contact, host-separated, solvent-separated, solvent-shared) [1,2,3,4,5,6,7,8,9,10]. Ion pair receptors also vary in the number of binding sites they possess, ranging from ditopic and tritopic structures [11] to more complex multitopic molecules [5]. Most such receptors have been designed to target alkali metal halides due to their prevalence in biological and environmental systems [7]. For example, special attention has been directed toward the development of receptors selective for lithium salts, driven by the widespread use of Li-ion batteries [4,12]. These compounds should rely on “hard” oxygen atoms for cation coordination and utilize hydrogen or halogen bonds for anion binding. Furthermore, ion pair receptors exhibit the potential of facilitating the recovery of other valuable materials from waste streams [4].

**Scheme 1:**
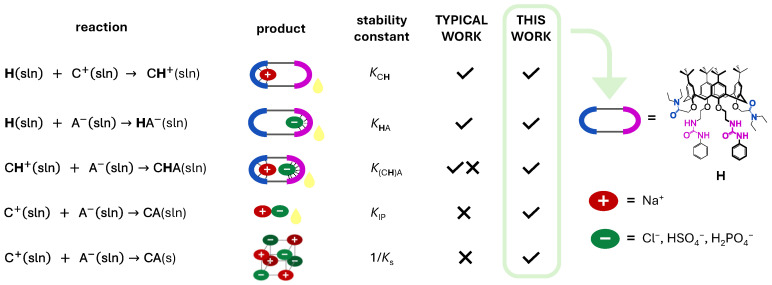
Thermodynamic model describing all reactions relevant to the investigation of cation-induced cooperativity in the binding of anions with the heteroditopic host in nonaqueous solvent, with differences between the scope of typical work in this field and this work. Legend: **H** = host; C^+^ = cation; A^−^ = anion; IP = ion pairing in solution; sp = solubility product.

In ion pair binding studies, the concept of cooperativity is frequently invoked [1,2,3,4,5,6,7]. Specifically, the binding of one ion type can influence the binding affinity of the resulting species for the other type of ion. This influence can be binary, whereby one ion does not bind unless the other is present (switch-on mechanism) [13,14,15]. Alternatively, it can modulate the receptor affinity for the second ion in a more subtle way, either enhancing or diminishing it, leading to positive (more often) or negative cooperativity [1,2,3,4,5,6,7,16]. The source of cooperativity is an electrostatic interaction between the bound ions and in allosteric effects that accompany the binding event. Both contributions are present in most cases, although it is rather difficult to discriminate and quantitatively ascertain each contribution. This issue was recently addressed by DFT calculations describing the binding of contact sodium halides to an aryl-triazole-ether macrocycle in dichloromethane [17]. It revealed that 70% of the positive cooperativity arose from the electrostatic effects, which unlike the allosteric contribution, were inversely proportional to the size of the halide anion. The latter work of Qiao et al. is also valuable, as it is a very rare example of an ion pair binding investigation where all possible thermodynamic equilibria in solution were included in the modeling (Scheme 1) [17].

In most ion pair binding studies, the binding to a host molecule (H) is quantified using one of two approaches [1,2,3,4,5,6,7,18]: (1) enhancement studies, where 1 equivalent of the cation (C^+^ in the form of salt with a bulky anion) is added to the host molecule, and the resulting mixture (often treated as a single inseparable CH^+^ species) is titrated with the desired anion (in the form of salt with a bulky cation); (2) Direct studies, where the solution of host is titrated with CA salt (ion pair) treated as an inseparable entity. The first approach provides reliable results only if *K*(CH^+^) is rather high (log *K* > 4), whereas the effect of the cation is significantly underestimated if this condition is not met. This is often acknowledged but rarely quantified, even in the most recent publications [19]. For instance, in the study by Munasinghe et al., apparent association constants were reported under conditions where only 20% of the receptor existed in the LiH^+^ form [15]. The second approach, which treats the ion pair as an inseparable entity, precludes distinguishing between the individual contributions of the cation and anion and the processes associated with each species. Moreover, the presence of free ions, even in small percentages, often requires dissolving the salt titrant in water or another polar solvent, which can affect the selectivity of the ion binding. [18,20]. Investigations of the influence of the solvent choice on the ion pair selectivity have also been carried out, highlighting that the solvent properties can modulate the ion binding behavior [21].

The primary challenges in ion pair binding studies are the formation of very stable solvated ion pairs and the low solubility of the corresponding salt in nonaqueous solvents, which are commonly used for these studies [3,4,8,22,23,24,25]. These factors are rarely quantified and are often merely noted. For instance, in the work by Tumcharern et al., the “lag” observed at the beginning of the NMR titration curve during the titration of the Na^+^ complex of an amide–thiourea calixarene with TBAOAc in MeCN was attributed to the strong ion pairing of NaOAc [26]. Similarly, Bregović et al. examined the binding of Na^+^ and F^−^ by a tryptophan–calixarene derivative in MeCN and reported strong ion pairing of NaF, which could not be quantitatively accounted for [27]. In the recent enhancement studies of alkali halide binding by a [2]catenane receptor, Tay et al. observed precipitation of the studied ion pairs in several cases [28]. Furthermore, Yang et al. utilized the strong ion pairing of NaF and its low solubility as a key factor for altering the cation selectivity in an ion pair receptor study. However, the processes involving NaF were not quantified [20].

An important class of receptors for various ions undoubtedly is that containing calixarenes [27,29,30,31,32,33,34,35,36,37]. These compounds studied as ion pair binders typically feature (thio)urea moieties (well-known for their strong anion-binding properties) [38,39,40,41,42] and ether, amide, or ester groups (which along with phenolic oxygen atoms of the calixarene scaffold coordinate various cations) [36,37,43,44,45,46]. In most cases, the cation-binding site is located at the narrow rim of the calixarene, while the anion-binding site is situated at the wider rim. These calixarenes have primarily been used for the recognition of alkali halides, where cations typically induced positive cooperativity [13,47,48,49,50,51]. This phenomenon is attributed to the rigidification of the calixarene structure upon cation binding [47]. (Thio)ureido calixarenes [39,52,53,54,55,56,57,58,59] and homooxacalixarenes [60,61,62,63,64,65] have been extensively investigated as anion binders. More recently, particularly with calix[6]arenes, these receptors have been employed in the selective recognition and transmembrane transport of various biologically relevant alkylammonium salts and zwitterions [14,66,67,68,69,70,71].

In this work, we conducted comprehensive ion pair recognition studies by a heteroditopic calixarene **H** in acetonitrile (Scheme 1). This receptor has been previously employed for anion binding [58] and pH-controlled supramolecular capsule formation, [57] and the related results inspired us to extend the studies of this supramolecular receptor. The primary objective of this work was to quantify the cation-induced enhancement of its anion-binding properties by fully elucidating thermodynamic equilibria taking place in the investigated solutions. Particular attention was directed to characterizing side processes linked to the ion pair recognition, specifically salt precipitation and free ion pair formation. This was achieved by using a combination of techniques, namely potentiometry, conductometry, ITC, and flame atomic emission spectroscopy (AES). For the investigation of anion binding with **H** and Na**H**^+^, we employed UV spectrophotometry, ITC, and ^1^H NMR titrations. In addition, the binding of NaCl by **H** was investigated via molecular dynamics (MD) simulations to gain insight into the structural features of the complexes and rationalize the observed positive cooperativity.

## 2. Results and Discussion

### 2.1. Complexation of Alkali Metal Cations with Host Calixarene in Acetonitrile

**Figure 1:**
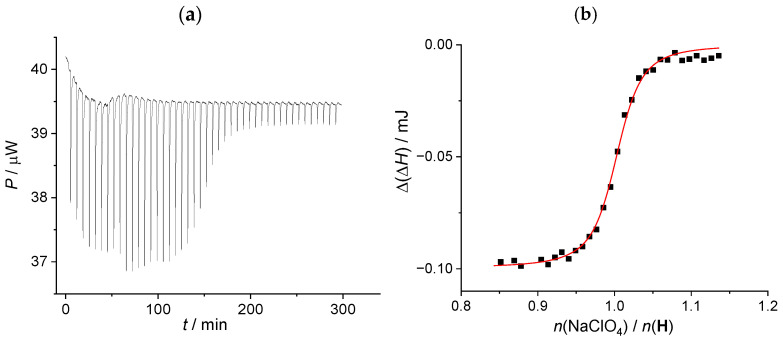
(**a**) Microcalorimetric titration of **H** 80% saturated with Na^+^ (*c*(**H**) = 1.66 × 10^−4^ mol dm^−3^, *c*(NaClO_4_) = 1.33 × 10^−4^ mol dm^−3^, *V*_0_ = 1.425 mL) with NaClO_4_ (*c* = 4.04 × 10^−4^ mol dm^−3^) in acetonitrile at 25 °C. (**b**) Dependence of successive enthalpy change on *n*(NaClO_4_)/*n*(**H**) ratio; ■ experimental; ―calculated.

The affinity of amide–urea calixarene derivative **H** towards alkali metal cations in acetonitrile was examined as the first step towards investigating the hypothesized ion-binding cooperativity. The goal was to find a cation that forms a strong complex with **H** with a stability constant that can be reliably determined. The microcalorimetric (ITC) titrations of receptor **H** with lithium, sodium, and potassium perchlorate indicated a very high affinity of **H** towards Na^+^ and Li^+^ (Figure 1 and Figure S2), while moderate stability was detected in the case of K^+^ (Figure S4, Table 1). All three cations formed complexes with 1:1 stoichiometry. The binding constant for the complexation of Li^+^ with **H** was too high for reliable direct determination (log *K* > 7). A displacement titration of K**H**^+^ with Li^+^ could not be applied to this end, due to the similar reaction enthalpies of the two complexes (Table 1). On the other hand, the stability constant of Na**H**^+^ species could be determined via a direct titration experiment (log *K* = 6.69); in spite of this, the related value was above the value usually considered to be reliably measurable via ITC (or UV; Figure S3). For this reason, a specific experimental procedure was applied to solve this task. Namely, the starting titrand solution contained the host **H** and 0.8 molar equivalents of NaClO_4_. In this way, the titration was focused on the conditions where the extent of the complexation equilibrium significantly depended on the composition of the solution (close to the equivalence point), and the corresponding curve provided enough information for determination of the stability constant.

The binding of all three cations by **H** was enthalpy-driven, with the formation of Na**H**^+^ featuring the most favorable Δ_r_*H*°. On the other hand, the entropic term (−*T*∆_r_*S*°) was significantly less favorable in the case of K^+^ compared to Li^+^ (Table 1). Similar behavior was observed in the case of lower-rim carbonyl-calix[4]arene (secondary amide, ketone, tertiary amide) derivatives (Figure S5) [36,44,72,73]. It is interesting to note that **H** has almost 4 orders of magnitude lower affinity towards Li^+^, Na^+^, and K^+^ than its phenanthridine analogue [44]. This is almost completely caused by the difference in standard complexation entropy, which indicates extensive variation in solvation of the calixarene receptors.

Based on the above elaborated results, we chose a sodium cation for further research into ion pair binding.

### 2.2. Solubility of Selected Sodium Salts and Related Ion Pairing in Acetonitrile

We selected three anions (chloride, hydrogen sulfate, and dihydrogen phosphate) to investigate the ion pair binding by **H**. The binding of these anions by **H** has been recently thermodynamically characterized [58]. The anions with weak to modest affinity for **H** were deliberately chosen, as it was assumed that Na^+^ binding by **H** would have a cooperative effect on the anion binding.

The next step in exploring the details of ion pair complexation was to determine several key thermodynamic parameters related to the salts (NaCl, NaHSO_4_, NaH_2_PO_4_) in acetonitrile, specifically their solubility (*s*), solubility product (*K*_s_), and related ion pair stability constant (*K*_IP_). Although this may seem a simple task, significant challenges emerge from their quite extreme values, as well as the fact that the processes are interconnected, i.e., the extent of one affects the others. Various methods were, thus, employed in order to obtain reliable data, and each approach is discussed in detail below.

#### 2.2.1. Sodium Chloride

The chemical equilibria taking place can be presented by Equations (1) and (2), with the accompanying equilibrium constants given by Equations (3) and (4).

(1) $$\mathrm{NaCl}(s)\;⇄\;{\mathrm{Na}}^{+}\left(sln\right)+{\mathrm{Cl}}^{-}\left(sln\right)$$

(2) $${\mathrm{Na}}^{+}\left(sln\right)+{\mathrm{Cl}}^{-}\left(sln\right)\;⇄\;\mathrm{NaCl}(\mathrm{sln})$$

(3) $$K_{s}(\mathrm{NaCl})=\;{\left[{Na}^{+}\right]}_{sat}\;\cdot \;{\left[{Cl}^{-}\right]}_{sat}$$

(4) $$K_{\mathrm{IP}}(\mathrm{NaCl})=\left[NaCl\right]\;/\;(\left[{Na}^{+}\right]\;\cdot \;\left[{Cl}^{-}\right])$$

Two methods (A and B below) were applied in order to determine the values of the above defined equilibrium constants and to confirm that indeed the assumed processes occurred in the investigated solutions (Table 2). Both approaches relied on the use of an ion-selective electrode for Na^+^ (Na-ISE), i.e., the potentiometric determination of the sodium cation concentration.

##### Method A

**Figure 2:**
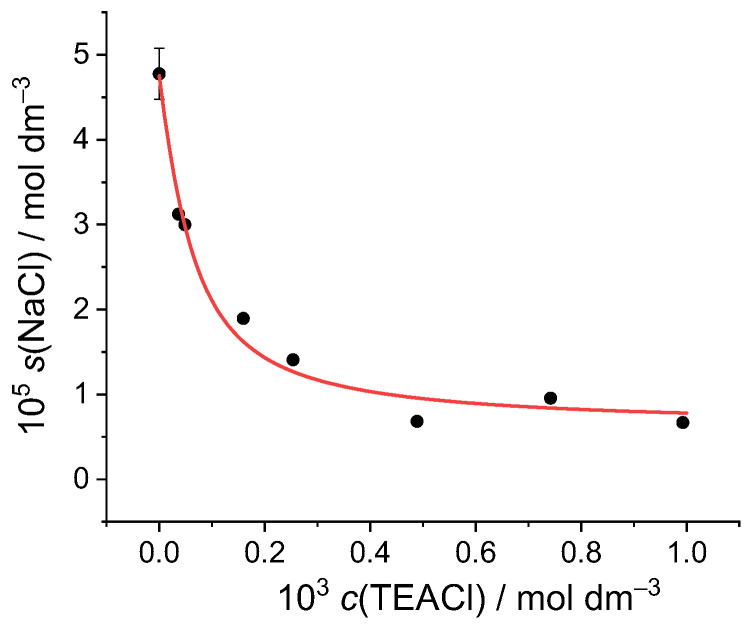
Solubility of NaCl in acetonitrile at different concentrations of TEACl at 25 °C; ● experimental; ― calculated using method A.

Method A included the preparation of saturated solutions of NaCl in pure acetonitrile and in the solutions of TEACl in this solvent. Once saturated, these solutions were filtered, the acetonitrile was evaporated, and the residue was dissolved in aqueous buffer of a much smaller volume than the volume of the filtered acetonitrile solution. The total concentration of Na^+^ in the prepared aqueous solution could be measured potentiometrically and used to calculate the total sodium present in the saturated acetonitrile solution (Figure 2). This is equal to the sum of the concentrations of the free sodium cation and ion pair (NaCl) in the saturated solution, as defined by Equation (5):

(5) $$s\left(NaCl\right)={\left[{Na}^{+}\right]}_{sat}+{\left[NaCl\right]}_{sat}$$

If there are no other salts in the saturated solution of NaCl in MeCN, Equation (5) can be rewritten using Equations (3) and (4):

(6) $$s{\left(NaCl\right)}_{pure}=\sqrt{K_{s}}+K_{s}\cdot \;K_{IP}$$

This value could be reliably determined, although as it can clearly be seen from Equation (6), it is defined by two parameters that cannot be calculated solely using this information (*s*(NaCl)_pure_). However, the extent of the underlying equilibria (Equations (1) and (2)) is modified by the addition of chloride (TEACl), which in turn affects the total solubility as defined by Equation (7), obtained using Equations (3)–(5):

(7) $$s{\left(NaCl\right)}_{with\;TEACl}=0.5\;\cdot \;\left(2K_{s}K_{IP}-c\left(TEACl\right)+\sqrt{{c\left(TEACl\right)}^{2}+4K_{s}}\right)$$

By processing the dependence of the NaCl solubility in acetonitrile on the total chloride concentration using Equation (7) (details given in Section 3), the *K*_s_ and *K*_IP_ values could be calculated (Table 2).

##### Method B

**Figure 3:**
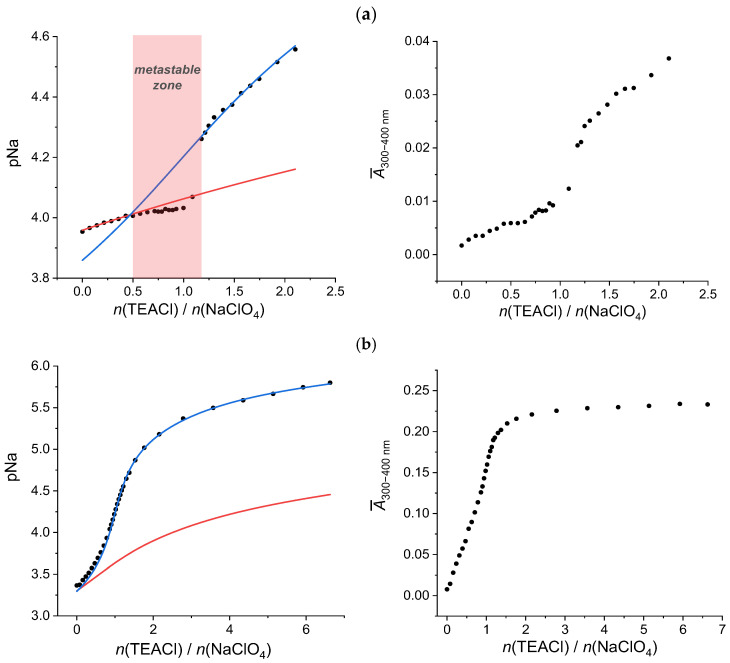
Potentiometric–turbidimetric titration of NaClO_4_ with TEACl in acetonitrile solution of TBAClO_4_ (*c* = 1 × 10^−2^ mol dm^−3^, *V* = 25 mL): (**a**) *c*(NaClO_4_) = 1.10 × 10^−4^ mol dm^−3^, *c*(TEACl) = 9.80 × 10^−3^ mol dm^−3^; (**b**) *c*(NaClO_4_) = 5.00 × 10^−4^ mol dm^−3^, *c*(TEACl) = 9.80 × 10^−3^ mol dm^−3^. For the description of the experimental potentiometric data (●), two models were used—including NaCl precipitation (―); omitting NaCl precipitation (―). Part of the titration data marked in red was not used in the calculation of the thermodynamic parameters.

The other method used for the determination of *s*, *K*_s_, and *K*_IP_ for NaCl in acetonitrile included the potentiometric (ISE for Na^+^) titration of NaClO_4_(sln) with TEACl(sln) coupled with a turbidimetric evaluation of the precipitation onset (Figure 3). The experiment was conducted at constant ionic strength to ensure that the thermodynamic parameters indeed remained constant throughout the titration.

The titrations of NaClO_4_(sln) with TEACl(sln) were performed using NaClO_4_ solutions at two different concentrations (Figure 3 and Figure S9). When the lower concentration was used, two regimes of pNa change were observed—before and after the start of precipitation. The selection of the appropriate set of equations describing the equilibrium was defined by the relationship between [NaCl] and the *K*_s_∙*K*_IP_. Namely, for [NaCl] < *K*_s_∙*K*_IP_, the ion pairing constant and total concentration of salts determine the free sodium ion concentration. On the other hand, if [NaCl] > *K*_s_∙*K*_IP_, and solid NaCl appears, the solubility product becomes the decisive factor. The validity of the applied data processing procedures was confirmed by the quality of fit and turbidimetric results. Namely, the precipitate formation could not easily be detected by the naked eye, although the decrease in solution transmittance enabled us to monitor the solid salt particles emerging in the system.

When no solid NaCl is present in the solution, the following equations define the mass balance:

(8) $$c_{{Na}^{+}}=\left[{Na}^{+}\right]+\left[NaCl\right]$$

(9) $$c_{{Cl}^{-}}=\left[{Cl}^{-}\right]+\left[NaCl\right]$$

where $c_{{\mathrm{Na}}^{+}}$ and $c_{{\mathrm{Cl}}^{-}}$ are analytical concentrations of NaClO_4_ and TEACl, respectively. From Equation (6), the ion pairing constant can be defined as a function of *s*(NaCl)_pure_ (in the following text, this value will be denoted by *s* for simplicity) and *K*_s_. Combining Equations (4), (8) and (9) and solving the resulting relation provides the free Na^+^ concentration as a function of the total Na^+^ and Cl^−^ concentrations:

(10) $$\left[{Na}^{+}\right]=c_{{Na}^{+}}-\frac{\left(K_{IP}\left(c_{{Na}^{+}}+c_{{Cl}^{-}}\right)+1\right)-\sqrt{{\left(K_{IP}\left(c_{{Na}^{+}}+c_{{Cl}^{-}}\right)+1\right)}^{2}-4{K_{IP}}^{2}c_{{Na}^{+}}c_{{Cl}^{-}}}}{2\;\cdot \;K_{IP}}$$

When the NaCl precipitate is also present in the system, the mass balance equations yield the following expression:

(11) $$c_{{Na}^{+}}-c_{{Cl}^{-}}=\left[{\mathrm{Na}}^{+}\right]-\left[{\mathrm{Cl}}^{-}\right]$$

In combination with Equation (3), this relation enables the derivation of the free (dissolved) Na^+^ concentration as a function of *K*_s_:

(12) $$\left[{Na}^{+}\right]=0.5\cdot \left(\left(c_{{Na}^{+}}-c_{{Cl}^{-}}\right)+\sqrt{{\left(c_{{Na}^{+}}-c_{{Cl}^{-}}\right)}^{2}+4K_{s}}\right)$$

Therefore, the two distinct parts of the dataset obtained via the potentiometric titration of NaClO_4_ with TEACl (Figure 3a) were processed simultaneously, albeit by two models, depending on whether the NaCl precipitate was formed (Equation (12)) or not (Equation (10)). This procedure provided the values of *s* and *K*_s_ (Table 2), which allowed the calculation of *K*_IP_ using Equation (6). On the other hand, when higher concentrations of salts were used, the precipitation of NaCl occurred immediately with the first addition of the titrant, as in the cases of the titrations presented in Figure 3b and Figure S9. Consequently, all pNa values measured in these cases obeyed Equation (12). This demanded the parameter *s*(NaCl) to be kept constant (at the value obtained via titration shown in Figure 3a) during the data processing, while *K*_s_ was the only adjustable parameter. The Excel Solver tool was used for all optimization procedures within method B, and the obtained values are provided in Table 2. The results gained by both methods were in reasonable agreement, confirming the validity of the related hypotheses. The solubility of NaCl in MeCN determined in this study was approximately twice as high as the value reported by Coetzee (3 × 10^−5^ mol dm^−3^) [22]. Additionally, the value for NaCl in MeCN at 25 °C obtained here aligns closely with the value of p*K*_s_ = 8.3 determined by Kolthoff and Chantooni through conductance measurements of a saturated NaCl solution [74].

#### 2.2.2. Sodium Hydrogen Sulfate

The solubility of NaHSO_4_ in pure acetonitrile was determined via flame AES (Table 3). It is worth noting that flame AES was applied in other cases but the solubilities of other sodium salts were too small for reliable determination using this method. Due to the greater solubility of NaHSO_4_ in MeCN, with respect to NaCl, more direct methods of obtaining ion pairing constants for NaHSO_4_ could be used, including conductometry (method C) and microcalorimetry (method D) methods.

##### Method C

When NaClO_4_ was titrated with TBAHSO_4_ in MeCN, at concentrations low enough to prevent NaHSO_4_ precipitation, a drop in the conductivity (Figure S10) was detected and ascribed to the ion pairing of NaHSO_4_. The ion pairing of TBAClO_4_, TBAHSO_4_, and NaClO_4_ was considered negligible in accordance with several studies (*K*_IP_ < 25 mol^−1^ dm^3^) [75,76,77]. The obtained conductometric titration curve was processed as described in the work by Barišić et al. [78]. The resulting *K*_IP_ value demonstrated that the ion pairing of NaHSO_4_ in MeCN is significantly more favorable than for NaCl. This finding was somewhat unexpected, especially if the anions sizes are considered. However, many previously reported examples indicate that ion pairing thermodynamics is influenced by multiple factors beyond the ion size (e.g., in MeCN *K*_IP_(LiBr) >> *K*_IP_(LiClO_4_) but *K*_IP_(NaI) << *K*_IP_(NaClO_4_)) [76]. During the fitting procedure, the molar ionic conductivities for Na^+^, ClO_4_^−^, and TBA^+^ were kept constant using the literature values [79], whereas *λ* for HSO_4_^−^ had to be treated as an adjustable parameter in the course of the regression analysis (no literature value was found). In order to test the reliability of the obtained value, *λ*_∞_(HSO_4_^−^) was also calculated from the conductivity measurement of TBAHSO_4_ solutions (Figure S11). Indeed, the value of *λ*_∞_(HSO_4_^−^) attained in the latter manner (58.5 S cm^2^ mol^−1^) was in very good agreement with the one obtained from the titration experiment (57.3 S cm^2^ mol^−1^; Figure S10).

The *K*_IP_ for NaHSO_4_, determined via conductometry, combined with its solubility value derived from the flame AES, enabled the calculation of its solubility product using Equation (6). The resulting value, 5.8 × 10^−8^ mol^2^ dm^−6^, was approximately one order of magnitude higher than the solubility product obtained for NaCl. This result was in line with the observed difference between the solubilities of these two salts.

##### Method D

**Figure 4:**
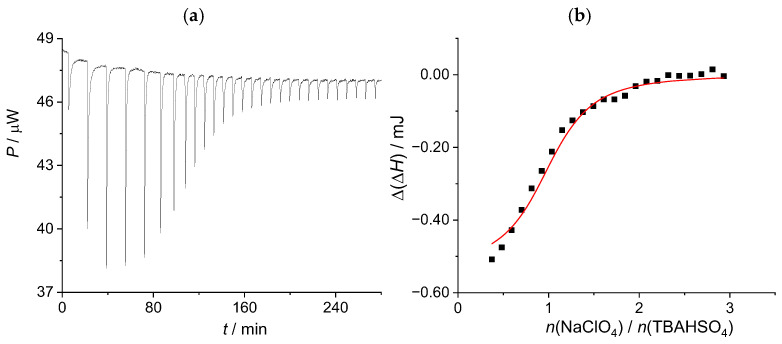
(**a**) Microcalorimetric titration of TBAHSO_4_ (*c* = 4.99 × 10^−4^ mol dm^−3^, *V*_0_ = 1.425 mL) with NaClO_4_ (*c* = 7.51 × 10^−3^ mol dm^−3^) in acetonitrile at 25 °C. (**b**) Dependence of successive enthalpy change on *n*(TBAHSO_4_)/*n*(NaClO_4_) ratio; ■ experimental; ― calculated.

The second method we employed to evaluate the ion pairing constant for NaHSO_4_ was ITC. The titration curve obtained via the titration of NaClO_4_ with TBAHSO_4_ in MeCN (Figure 4) enabled the calculation of the corresponding reaction enthalpy (−7 kJ mol^−1^) and ion pairing constant for NaHSO_4_ (Table 3). The highly favorable reaction entropy related to ion pairing (−*T*·Δ_r_*S*° = −19 kJ mol^−1^) most likely resulted from a significantly lower number of solvent molecules included in the solvation of NaHSO_4_ than in the solvation of free ions. The ITC value for *K*_IP_(NaHSO_4_) was in good agreement with the one obtained from the conductometric measurements (Table 3). Furthermore, this value enabled the calculation of the solubility product for NaHSO_4_ (2.6 × 10^−8^ mol^2^ dm^−6^), as in method C.

#### 2.2.3. Sodium Dihydrogen Phosphate

The solubility, the solubility product, and the ion pairing constant for NaH_2_PO_4_ in acetonitrile were evaluated (Table 4) using two potentiometric methods (E and F; see Section 3). These methods were similar to methods A and B, respectively, although the dimerization of dihydrogen phosphate in MeCN [38] caused the underlying equations to be more complicated.

##### Method E

The solubility of NaH_2_PO_4_ in an acetonitrile solution containing TBAH_2_PO_4_ is given by the following equation:

(13) $$\;s\left({\mathrm{NaH}}_{2}{\mathrm{PO}}_{4}\right)={\left[{H_{2}{\mathrm{PO}}_{4}}^{-}\right]}_{sat}+2{\left[{\left(H_{2}{\mathrm{PO}}_{4}\right)}_{2}^{2-}\right]}_{sat}+{\left[{\mathrm{NaH}}_{2}{\mathrm{PO}}_{4}\right]}_{sat}-c(TBAH_{2}{\mathrm{PO}}_{4})$$

Using Equations (S5)–(S9) and Equation (13), the solubility of NaH_2_PO_4_ (*s*) in the presence of TBAH_2_PO_4_ can be written as an implicit function of *c*(TBAH_2_PO_4_) (represented as *c* in Equation (14)), *K*_s_, *K*_IP_, and *K*_dim_:

(14) $$\begin{array}{c} s^{3}+s^{2}\cdot \left(c-3K_{s}K_{IP}\right)+s\cdot \left(3{K_{s}}^{2}{K_{IP}}^{2}-K_{s}\cdot \left(1+2cK_{IP}\right)\right)+\\ +{{\;K}_{s}}^{2}\cdot \left(K_{IP}-2K_{dim}+c{K_{IP}}^{2}\;\right)-{K_{s}}^{3}{K_{IP}}^{3}=0 \end{array}$$

The fitting of the experimentally obtained solubilities of NaH_2_PO_4_ in acetonitrile with different concentrations of TBAH_2_PO_4_ by Equation (14) enabled the calculation of the desired physicochemical quantities regarding NaH_2_PO_4_ in MeCN (Figure S12, Table 4). It is noteworthy that the 20-fold lower solubility of NaH_2_PO_4_ compared to NaCl arises from a specific type of partial compensation; the *K*_IP_ for NaH_2_PO_4_ is ≈6 orders of magnitude larger than the one obtained for NaCl, and *K*_s_ for dihydrogen phosphate salt is ≈5 orders of magnitude smaller than *K*_s_(NaCl).

##### Method F

**Figure 5:**
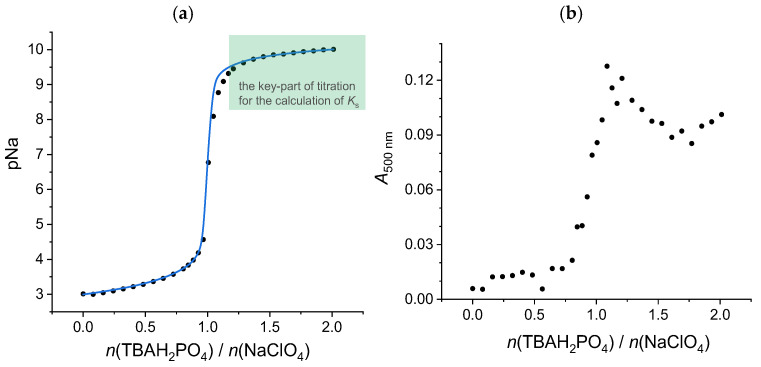
Potentiometric–turbidimetric titration of NaClO_4_ (*c* = 1.01 × 10^−3^ mol dm^−3^) in an acetonitrile solution of TBAClO_4_ (*c* = 1 × 10^−2^ mol dm^−3^, *V* = 25 mL) with TBAH_2_PO_4_ (*c*_2_ = 1.0 × 10^−2^ mol dm^−3^): (**a**) experimental potentiometric data (●) were fitted using method F (―); (**b**) absorbance increase observed upon the addition of TBAH_2_PO_4_ indicated the precipitation of NaH_2_PO_4_.

The second method used for the determination of *K*_s_ and *K*_IP_ for NaH_2_PO_4_ in acetonitrile was the simultaneous potentiometric–turbidimetric titration of NaClO_4_(sln) with TBAH_2_PO_4_(sln) (Figure 5 and Figure S14, Table 4) in combination with the value of *s*(NaH_2_PO_4_) obtained using method E. This method was very similar to method B, with the inclusion of dihydrogen phosphate dimerization in the model being the only difference (Table S2). The corresponding model was defined in the HySS program [80] to calculate pNa values during the titration experiment, with the *K*_s_ values varied to ensure the calculated pNa values closely matched the experimental ones. In this procedure, we were primarily focused on the section of the titration curve most sensitive to variations in *K*_s_ (highlighted in green in Figure 5 and Figure S14). The resulting value for *K*_s_ (−log *K*_s_ = 13.7, Table 4) was relatively close to the one obtained using method E. The ion pairing constant for NaH_2_PO_4_ could not be evaluated solely from the potentiometric titration data. However, for a saturated solution of NaH_2_PO_4_ in pure MeCN, the value of *K*_IP_ could be derived (see SI):

(15) $$K_{IP}=\left(s-{\left[{Na}^{+}\right]}_{sat}\right)/{\;K}_{s}$$

The concentration of [Na^+^] in the saturated solution of NaH_2_PO_4_ was determined using an implicit function dependent on *K*_s_ and *K*_dim_ (details of the derivation in SI):

(16) $$\;64K_{dim}^{2}\cdot {\left[{Na}^{+}\right]}^{6}-256K_{s}^{2}K_{dim}^{3}\cdot {\left[{Na}^{+}\right]}^{3}-64K_{s}^{2}K_{dim}^{2}\cdot {\left[{Na}^{+}\right]}^{2}+256K_{s}^{4}K_{dim}^{4}=0$$

Using this approach, the determined value of the ion pairing equilibrium constant was in a good agreement with the value obtained using method E (Table 4). Due to the higher reliability and accuracy of the data collected using method F, we used these data in the further studies discussed in this work.

### 2.3. Cooperativity in Binding of Sodium Ion Pairs by Host Calixarene in Acetonitrile

**Scheme 2:**
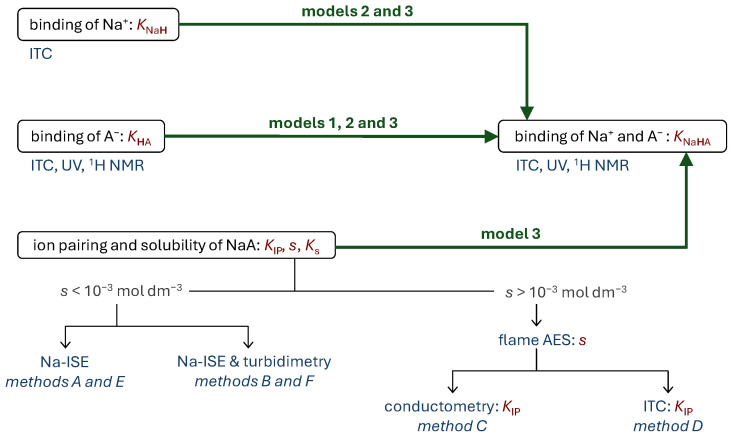
Workflow of the experimental determination of the thermodynamic data for all relevant processes encountered in this research.

**Figure 6:**
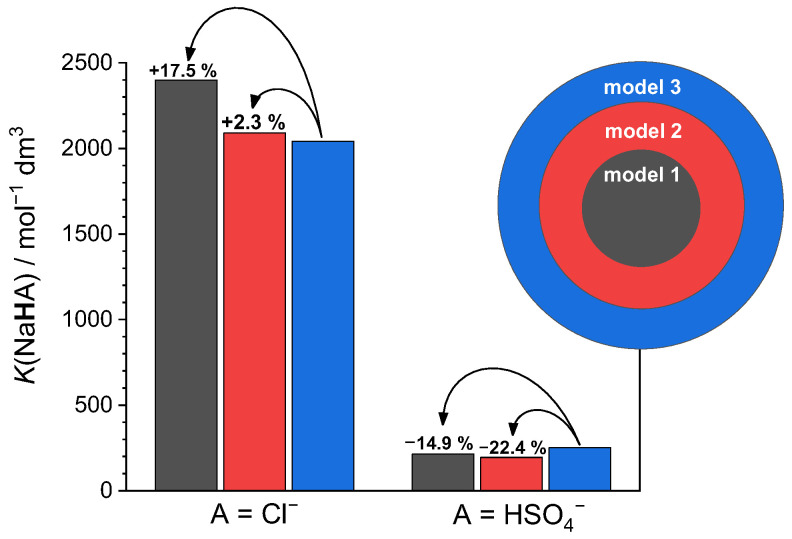
Comparison of the affinities of Na**H**^+^ for Cl^−^ and for HSO_4_^−^ in MeCN at 25 °C calculated from the results of the corresponding UV spectrophotometric titrations using different models (sets of thermodynamic equilibria in the solution, whereby model 3 contains all characterized equilibria, model 2 lacks ion pairing of NaA, and model 1 lacks ion pairing of NaA and treats Na**H**^+^ as an inseparable species).

By using the data discussed above, related to the behavior of the host as the single-ion receptor and the thermodynamic parameters for side processes, we were able to design the titration experiments and describe the studied system with a high level of scrutiny. The analysis of the respective titration data was carried out by considering all processes relevant for the studied systems, a difficult task that is rarely tackled and had to be adjusted for each system studied in this work. The workflow of the related experimental procedures is presented in Scheme 2, and the results obtained by applying them are summarized in Table 5 and Table 6, and Figure 6.

#### 2.3.1. Sodium Chloride

**Figure 7:**
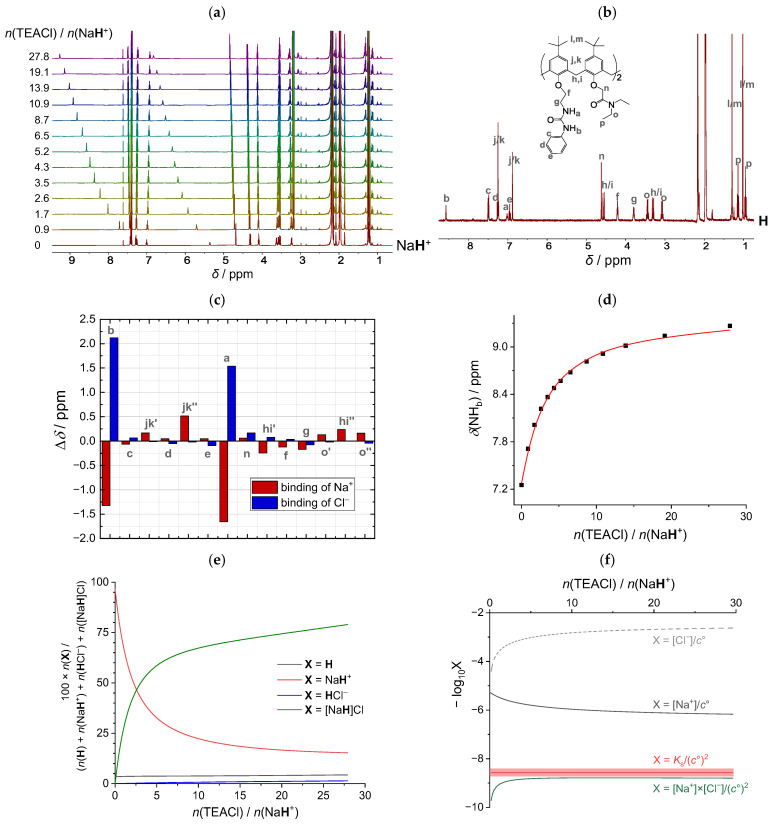
^1^H NMR spectroscopy titration of Na**H**ClO_4_ (*c* = 1.68 × 10^−4^ mol dm^−3^, *V*_0_ = 500 μL) with TEACl (*c* = 7.32 × 10^−3^ mol dm^−3^) in CD_3_CN at 25 °C. (**a**) ^1^H NMR spectra acquired during the titration. (**b**) Assignment of proton signals for receptor **H**. (**c**) Comparison of Δ*δ* changes for signals of protons at **H** during the binding of Na^+^ (*δ*(1 molEq Na^+^) − *δ*(free **H**)) and Cl^−^ (*δ*(28 molEq Cl^−^) − *δ*(1 molEq Na^+^)). (**d**) Experimental (■) and calculated (―) chemical shifts for ureido protons at Na**H^+^**. Calculation was performed with the approximation that all **H** is present in the form of inseparable Na**H**^+^ and that the only existing process is the complexation of Na**H**^+^ with Cl^−^ (model 1 in Figure 6). (**e**) Distribution of **H** and its complexes with Na^+^ or Cl^−^ during the titration with TEACl (calculated with HySS program) using the complete model demonstrated in Table S4, with log *K*(**H**Cl^−^) = 2.06 and log *β*(Na**H**Cl) = 10.0. (**f**) Simulation of distribution of free Na^+^ and Cl^−^ ions using the complete model (Table S4), as well as experimentally determined *K*_s_ for NaCl (Table 2). Results of simulation show that the product of concentrations of free Na^+^ and Cl^−^ is below *K*_s_. The difference between the values of *K*_s_ obtained using two methods (Table 2) was also taken into account (width of red line).

In order to explore the cooperativity within the binding of NaCl by calixarene **H**, the ^1^H NMR titration of the solution of **H** and NaClO_4_ (*n*/*n* = 1) with TEACl was performed (Figure 7). The concentrations of the reactants were low enough to ensure the lack of any precipitation event, i.e., the product of concentrations of free Na^+^ and Cl^−^ was less than *K*_s_ (log *K*_s_ = –8.7) throughout the titration (Figure 7f). Slow exchange kinetics (relative to the NMR timescale) was detected in the case of Na^+^ binding by **H**, while the complexation of Cl ^–^ with Na**H**^+^ featured a fast exchange regime. The most pronounced (downfield) shifts induced by the complexation were detected for urea protons (Figure 7c). The fitting of the chemical shifts of proton signals from **H** was done with the approximation that all **H** is present in the form of Na**H**^+^ at the beginning of the titration and that the only process is the complexation of Na**H**^+^ with Cl^−^ (Figure 7d). The elaborate model of binding could not be applied in this case due to the combination of slow and fast exchange kinetics. The resulting successive complexation constant for the formation of ternary complex Na**H**Cl from Na**H**^+^ (log *K* = 3.27, Table 5) was about one order of magnitude larger than the stability constant characterizing the binary complex **H**Cl^−^ [58], pointing out a significant positive cooperativity of the sodium cation on the binding of chloride by **H**.

The UV spectrophotometric curves related to the titrations of the mixture of **H** and NaClO_4_ (*n*/*n* = 1) with TEACl (Figure S15) could be processed by applying a complete series of thermodynamic equilibria (Table S4). This included the formation of Na**H**^+^(sln), **H**A^−^(sln), NaA(sln), and Na**H**A(sln). Characteristic spectra for **H**, Na**H**^+^, and **H**Cl^−^ were fixed during the fitting process using the values attained via independent experiments (titration of **H** with Na^+^ and **H** with Cl^−^). A rather similar value of the stability constant for Na**H**Cl was obtained both by including the ion pairing in the model and by ignoring it (Table 5). This was in accordance with the very low percentage of ions present in the form of ion pairs during the titration (Figure S15e,f). When the simplest model (treating the Na**H**^+^ as an “inseparable” species, with ion pairing ignored) was applied, a 17% higher value for *K*(Na**H**Cl) was obtained. Therefore, in the case of determining the cooperative effect of the cation on the interaction of **H** with Cl^−^ in MeCN, a more pronounced error is introduced by not incorporating the Na**H**^+^ formation equilibrium in the fitting model than by neglecting the ion pairing of NaCl (Figure 6). However, both simplified models resulted in *K*(Na**H**Cl) values very close to the value determined by the model comprising all processes occurring in the investigated solutions, justifying the use of a simplified binding model for processing the microcalorimetric titration of Na**H**^+^ with Cl^−^ (Figure S16). This yielded a value of *K*(Na**H**Cl) in good agreement with the ones obtained via NMR and UV. Interestingly, without preorganization of its binding cavity with Na^+^, the leading thermodynamic driving force for chloride binding with calixarene **H** was the reaction entropy (−*T*∆_r_*S*° = −9 kJ mol^−1^) [58]. The entropically favorable displacement of the acetonitrile molecule from **H** upon chloride binding, as revealed by the MD simulations (Table 7), is likely to account for this observation. On the contrary, the binding of Cl^−^ by sodium complex Na**H**^+^ was mainly enthalpically driven (Table 6), which also aligns with the MD study results, as discussed below.

**Figure 8:**
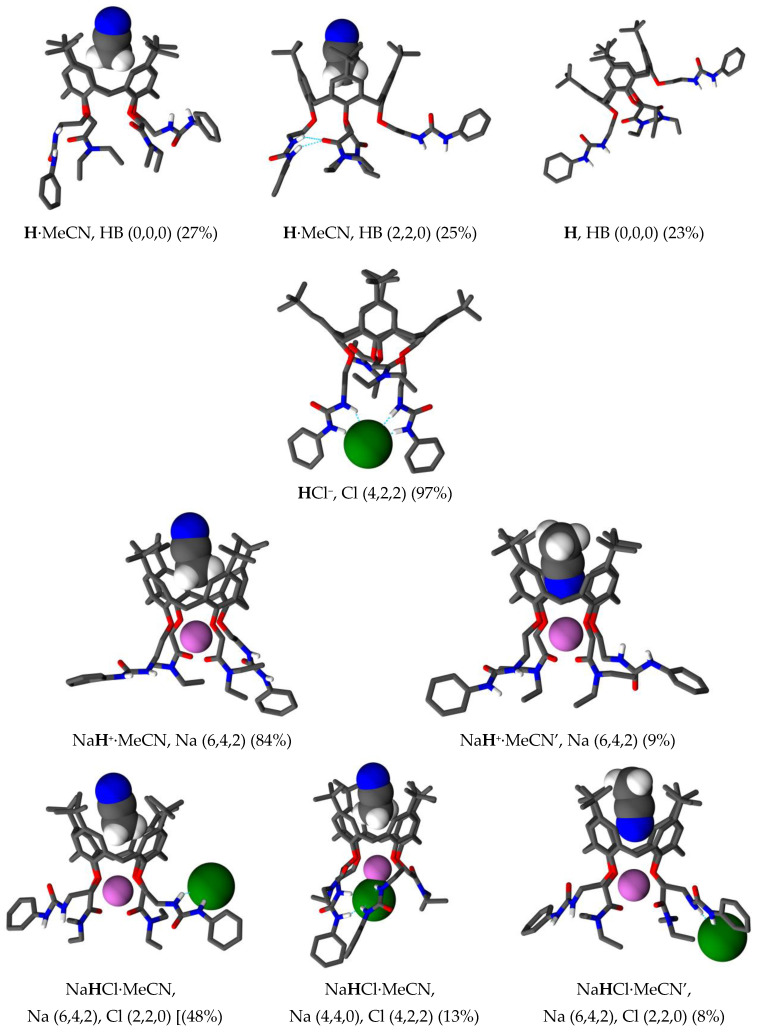
Representative structures of **H**, **H**Cl^−^, Na**H**^+^, and Na**H**Cl in MeCN obtained via MD simulations (50 ns, step = 1 ps, 298.15 K, 1 bar) with the abbreviated coordination (Na, Cl) or intramolecular hydrogen bonds (HB) patterns and the corresponding percentages of simulation time. Details about the Na, Cl, and HB patterns for these structures are given in Table S7. Legend: Na (total-O, ether-O, amide-O); Cl (total-NH, urea-1, urea-2); HB (total-NH, total with amide-O, total with urea-O). For representative structures of **H**Cl^−^, Na**H**^+^, and Na**H**Cl, the HB coordination pattern is equal to (0,0,0). All hydrogens, except those belonging to urea, were omitted for clarity.

To gain an insight into the structural features of the ternary Na**H**Cl complex and its differences from the relevant binary complexes Na**H**^+^ and **H**Cl^−^, as well as to explore potential structural reasons for the experimentally observed positive cooperativity of chloride binding by **H** in the presence of Na^+^, we conducted MD simulations of these complexes (and the free host) in MeCN at 25 °C. Representative molecular structures of the most populated clusters of free calixarene (**H**) and its ion complexes, classified by solvent inclusion and coordination patterns (intramolecular hydrogen bonds and Na^+^ or Cl^−^ coordination), were determined via a principal component analysis (PCA) on a coordination matrix (details in Section 3). The results are shown in Figure 8, and details of the systematic structural analysis are provided in Table S6.

Receptor **H** features two amide carbonyl oxygens (amide-O) and two urea moieties capable of forming intramolecular hydrogen bonds (HB). Indeed, the crystal structure of **H**·4CH_3_CN revealed that one amide-O forms two intramolecular hydrogen bonds with a urea moiety, while one CH_3_CN molecule is incorporated within the calixarene basket [57]. Consequently, we classified the MD results for free **H** in MeCN based on the inclusion of a solvent molecule and the presence of intramolecular hydrogen bonds. The latter was quantitatively represented using the HB coordination matrix (*x*, *y*, *z*), where *x*, *y*, and *z* indicate the numbers of intramolecular hydrogen bonds for the total, with amide-O, and with urea-O, respectively. The three most abundant clusters of structures for **H** in MeCN, along with their contributions to the simulation time (%), were as follows: **H**·MeCN, HB (0,0,0) (27%), **H**·MeCN, HB (2,2,0) (25%), and **H**, HB (0,0,0) (23%). In two clusters, MeCN was included in the calixarene basket via the CH_3_ end, forming a slightly flattened cone shape. The second most abundant cluster featured two intramolecular hydrogen bonds, with one amide oxygen coordinated with both NH groups of one urea moiety, as observed in the crystal form of **H**. The third cluster displayed a significantly flattened cone conformation due to the absence of the MeCN inclusion (Table S6).

For **H**Cl^−^, almost all structures (97%) obtained via MD belonged to the cluster **H**Cl^−^, Cl (4,2,2), HB (0,0,0), where the Cl matrix denotes the number of NH groups coordinating Cl^−^ (total, from 1st urea, from 2nd urea). In this conformation, chloride was coordinated by both urea moieties of calixarene **H**, which was consistent with the ^1^H NMR result (as shown in Figure S10 in our previous paper) [58] and is further discussed below. The favorable formation of four hydrogen bonds between the Cl^−^ ion and the urea functionalities in **H** enthalpically outweighed the unfavorable loss of intramolecular hydrogen bond(s) during anion binding (Table 7), facilitating the formation of **H**Cl^−^. The resulting complex adopted an even more flattened cone shape than **H**, HB (0,0,0).

For the Na**H**^+^ complex in MeCN, two clusters were most significant—Na**H**^+^·MeCN, Na (6,4,2), HB (0,0,0) (84%); and Na**H**^+^·MeCN’, Na (6,4,2), HB (0,0,0) (9%)—where the Na matrix indicates the number of oxygens coordinating Na^+^ (total, ether, amide). MeCN’ represents the MeCN included in the calixarene basket via the CN end. In both representative clusters, Na^+^ was coordinated with all four ether oxygens and both amide carbonyl oxygens. Most research studies on ureido derivatives of calixarenes have primarily focused on anion binding, leaving limited data on their coordination with metal cations. In this study, the urea oxygens were, therefore, analyzed for potential Na⁺ coordination. However, no Na⁺ coordination with urea oxygens was observed, likely due to their greater distance from the ether oxygens, which primarily coordinate Na⁺, compared to the amide oxygens. For Na⁺ to coordinate with the urea oxygens, they would need to bend, which is likely sterically hindered by the tertiary amides, meaning it does not occur. Similar steric hindrance effects have been observed with lower-rim tetra-tertiary amide derivatives of calixarenes, where the Na⁺ coordination in MeCN decreased upon substituent modification. Specifically, replacing a simple aliphatic chain with a triazolic group—both with a methylene spacer to the phenolic oxygens, as in the case of **H**—reduced the Na⁺ coordination number from 3.8 to 2.9. [36,81]. This finding partially supports the above explanation regarding lack of Na⁺ urea coordination in calixarene **H**. The inclusion of MeCN with the CH_3_ end in the calixarene basket of Na**H**^+^ was frequently reported previously [44,45,82,83]. The incorporation of Na^+^ into **H** (a) shifted its conformation from a flattened cone to a regular or very slightly flattened cone, depending on the solvent inclusion type (Table S6), and (b) increased the number of solvent molecules being exchanged inside the hydrophobic cavity of **H** during the simulation (Figure S19).

**Figure 9:**
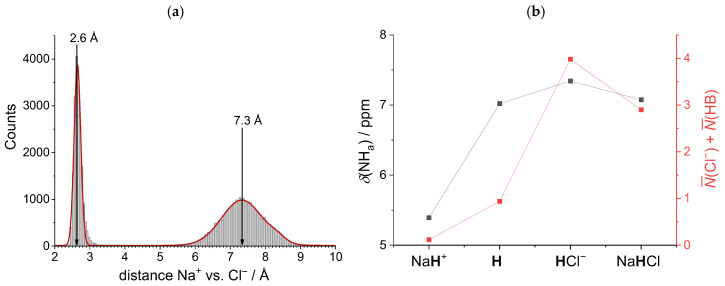
(**a**) Histogram showing the distribution of distances between Na⁺ and Cl^−^, as obtained from MD simulations of Na**H**Cl (50 ns, step = 1 ps, 298.15 K, 1 bar, in MeCN). Histogram bin size: 0.05 Å. Details of the distribution analysis are provided in SI. (**b**) Comparison of the experimental chemical shifts for urea protons (NH_a_) at **H** and its ion complexes with the corresponding total average number of NH contacts ($\overset{-}{N}\left({\mathrm{Cl}}^{-}\right)+\overset{-}{N}\left(\mathrm{HB}\right)$) for the same chemical species obtained using MD (conditions as in (**a**)).

The ternary Na**H**Cl complex in MeCN was represented by three clusters: Na**H**Cl·MeCN, Na (6,4,2), Cl (2,2,0), HB (0,0,0) (48%); Na**H**Cl·MeCN, Na (4,4,0), Cl (4,2,2), HB (0,0,0) (13%); and Na**H**Cl·MeCN’, Na (6,4,2), Cl (2,2,0), HB (0,0,0) (8%). In the most abundant cluster, Na^+^ was coordinated as in the Na**H**^+^ complex (with four ether oxygens and two amide oxygens), while Cl^−^ was bound to only one urea moiety. The average distance between Na^+^ and Cl^−^ was found to be 7.3 Å (Figure 9a and Figure S21), closely matching the 7.2 Å distance obtained via MD simulations for a solvent-shared ion pair of NaCl in a MeCN/DMF mixture (*x*(MeCN) = 0.75) [10]. In our system, Cl^−^ was partially solvated, while Na^+^ was fully surrounded by coordination atoms from the host molecule and the included acetonitrile. This coordination suggests that NaCl is a host-separated ion pair in the Na**H**Cl·MeCN, Na (6,4,2), Cl (2,2,0), HB (0,0,0) cluster. The increased distance between Na⁺ and Cl⁻ observed in the first cluster, when compared with the contact ion pair, was expectedly accompanied by a decrease in the strength of their ionic interactions (Figure S20b). In the second most abundant cluster, the Na^+^ coordination was reduced (the two amide oxygen atoms were excluded from the coordination sphere), while the Cl^−^ coordination increased. This adjustment might be attributed to steric constraints; it is geometrically challenging for both urea moieties to coordinate Cl^−^ while the other two amide oxygens coordinate the Na^+^ ion very closely. The small distance between the Na^+^ and Cl^−^ (2.6 Å, Figure 9a) aligns with that observed for the contact ion pair of NaCl (2.7 Å) obtained by MD in a MeCN/DMF mixture (*x*(MeCN) = 0.75) [10]. This distance also corresponds to the one measured when NaCl is bound as a contact ion pair to a heteroditopic calix[4]diquinone receptor in MeCN (2.5 Å) [84], and is shorter than the sum of crystallographic ionic radii of Na^+^ and Cl^−^ (2.83 Å) [85]. It is, therefore, clear that Na^+^ and Cl^−^ form a contact ion pair in the second representative cluster obtained for Na**H**Cl. The third most abundant cluster for Na**H**Cl in MeCN displayed the same coordination pattern as the first, differing only in the orientation of acetonitrile within the basket.

According to MD simulations, the positive cooperativity experimentally observed in the case of Na**H**Cl formation compared to **H**Cl^−^ could result from a combination of several factors (Table 7). The electrostatic attraction between Na^+^ and Cl^−^ enhances the affinity of Na**H**⁺ for Cl^−^ more significantly when these ions form a contact ion pair within calixarene **H**. During approximately 60% of the simulation time, the Na^+^ and Cl^−^ remained about 7.3 Å apart, forming a host-separated ion pair. A weaker electrostatic attraction was still present between them at this distance. The influence of this conformation became even more significant for the description of the Na**H**Cl structure when a 260 ns MD simulation was conducted (Figures S23 and S24). In general, the MD results are in line with the experimentally determined stability constants, considering both the electrostatic and coordination aspects. The complexes containing Na^+^ are capable of solvent molecule inclusion, which stabilizes these species. On the other hand, the coordination of the Cl^−^ ion with both urea groups (Figure 8) strongly favors a flattened cone conformation, which is not suitable for MeCN inclusion, adding to the difference in stability between **H**Cl^−^ and Na**H**Cl. Although the average number of hydrogen bonds with chloride is lower in the case of Na**H**Cl, this is compensated by the increase in intramolecular hydrogen bonds in Na**H**Cl compared to **H**Cl⁻. Finally, comparing the experimental chemical shifts of urea protons in all studied species (Na**H**^+^, **H**, **H**Cl^−^, and Na**H**Cl) with the total average number of NH contacts reveals a consistent trend—increases in both quantities for **H**Cl^−^, followed by a decrease when taking the ternary complex into account (Figure 9b). This can be explained by the dual role of urea protons, which act as donors coordinating chloride and forming intramolecular hydrogen bonds (primarily with amide oxygens).

#### 2.3.2. Sodium Hydrogen Sulfate

The sodium-induced cooperativity related to the binding of HSO_4_^−^ by calixarene **H** was initially evaluated via ^1^H NMR titration. An equimolar mixture of calixarene **H** and NaClO_4_ was titrated with TBAHSO_4_ (Figure S24). Strong downfield shifts of urea proton signals affirmed urea moieties as the primary binding site for HSO_4_^−^. The obtained titration curves (Figure S24b,c) were processed by applying a simple binding model where Na**H**^+^ was treated as inseparable and NaHSO_4_ ion pairing was ignored (model 1 in Figure 6). The resulting value of the apparent stability constant for the Na**H**HSO_4_ complex (log *K* = 2.45, Table 5) was 5 times larger than *K*(**H**HSO_4_^−^), again indicating positive cooperativity, although somewhat less pronounced than in the case of Cl^−^. It is interesting to note that the stability rates of the free ion pairs feature the opposite trend, with the ion pairing constant for NaHSO_4_ being 10 times greater than for NaCl. This was partly accounted for by the higher energetic cost of the desolvation upon binding for NaHSO_4_. Further, this result suggested that the conformational changes induced by Na^+^ binding better suited the binding of Cl^−^ than HSO_4_^−^.

The UV titration of an acetonitrile solution containing **H** and NaClO_4_ (*n*/*n* = 1) with TBAHSO_4_ induced hyperchromic shifts in the corresponding spectra (Figure S26). These shifts were accounted for by considering all relevant thermodynamic equilibria—namely, the formation of Na**H**^+^, **H**HSO_4_^−^, NaHSO_4_ (sln), and Na**H**HSO_4_—and by fixing the characteristic spectra for **H**, Na**H**^+^, and **H**HSO_4_^−^ according to the UV titration results described in our previous work [58]. The most informative part of the spectra was the one collected at wavelengths < 278 nm, where the molar absorbances of Na**H**^+^ and Na**H**HSO_4_ were significantly different (Figure S26c). The full thermodynamic model (model 3 in Figure 6) provided the value of the successive stability constant of the Na**H**HSO_4_ complex (Table 5). During the fitting procedure, it was also possible to refine the value of the ion pairing constant for NaHSO_4_, and the result was in accordance with the one obtained using independent methods (conductometry and ITC, Table 3). Ignoring the NaHSO_4_ ion pairing in the calculation of this stability constant resulted in a 22% reduction in *K*(Na**H**HSO_4_) (Figure 6). This more considerable change in the value of the ternary complex stability constant (in comparison to Na**H**Cl) obtained by using the simplified vs. complete model was in line with the significant extent of ion pair formation during the titration (Figure S26e,f). The application of the simplest thermodynamic model (model 1 in Figure 6) for the description of the spectrophotometric titration curve attained for the system of Na**H**^+^ + HSO_4_^−^ resulted in a 15% smaller value than that calculated using the full model.

The application of the full model of binding (model 3 in Figure 6) was not possible in the case of the corresponding NMR titration due to the combination of slow (Na**H**^+^) and fast (**H**HSO_4_^−^ and Na**H**HSO_4_) exchanges (similar as for the system Na**H**^+^ + Cl^−^). However, the related thermodynamic data obtained by other methods (Table S9) enabled us to calculate the concentrations of free Na^+^ and HSO_4_^−^ ions throughout the NMR titration. This ensured us that no precipitation of NaHSO_4_ was happening within the experimental conditions used for the NMR titration (Figure S24) and provided insight into the speciation during titration (Figure S24d), which was in line with the intricate spectral behavior observed via ^1^H NMR.

#### 2.3.3. Sodium Dihydrogen Phosphate

In order to explore the cooperative effect of Na^+^ on the binding of H_2_PO_4_^−^ by the receptor **H**, microcalorimetric titration of a mixture of **H** and NaClO_4_ (*n*/*n* = 1) with TBAH_2_PO_4_ was performed. However, the significant endothermic heat effects, produced until the equimolar addition of H_2_PO_4_^−^ (Figure S27), suggested that the precipitation of NaH_2_PO_4_ was taking place along with anion binding by Na**H**^+^, preventing further ITC studies. Namely, despite the rather low concentration of Na^+^ (0.2 mmol dm^−3^), the precipitation of NaH_2_PO_4_ was obviously the dominant process due to the very low value of *K*_s_ and the high value of *K*_IP_.

**Figure 10:**
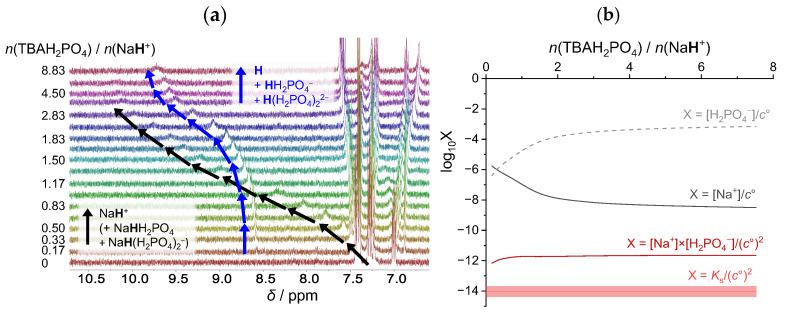
^1^H NMR spectroscopy titration of Na**H**ClO_4_ (*c* = 8.97 × 10^−4^ mol dm^−3^, *V*_0_ = 490 μL) with TBAH_2_PO_4_ (*c* = 1.46 × 10^−2^ mol dm^−3^) in CD_3_CN at 25 °C. (**a**) ^1^H NMR spectra acquired during titration. Complexation of both **H** and Na**H**^+^ species with H_2_PO_4_^−^ was detected but could not be quantified. (**b**) Simulation of ^1^H NMR titration of Na**H**ClO_4_ with TBAH_2_PO_4_ using model demonstrated in Table S10. Results of simulation show that the product of the concentrations of free Na^+^ and H_2_PO_4_^−^ is greater than *K*_s_ for NaH_2_PO_4_. More details about this experiment can be found in Figure S28.

The proton NMR titration of **H** and NaClO_4_ (*n*/*n* = 1) with TBAH_2_PO_4_ (Figure 10 and Figure S28) in MeCN provided deeper insight into the binding of H_2_PO_4_^−^ with the Na**H**^+^ complex. As mentioned earlier, the binding of Na^+^ by **H** was a process happening at a slow exchange rate when compared to the NMR scale, which meant that two sets of signals were detected. At the beginning of the NMR titration, almost all of the **H** was in the form of Na**H**^+^. The addition of H_2_PO_4_^−^ into the Na**H**^+^ solution caused a downfield shift of Na**H**^+^ urea–proton signals and a slow decrease in their intensities (Figure 10a). On the other hand, already at 0.17 eq. of added titrant, urea–proton signals belonging to free **H** appeared, and with the further addition of H_2_PO_4_^−^ their intensity grew, which was coupled with a downfield shift. If no formation of Na**H**(H_2_PO_4_)_x_^x−^ (x = 1 and/or 2) was present in this experiment, the proton signals assigned to Na**H**^+^ would decrease as H_2_PO_4_^−^ were added. After reaching equivalence, all Na^+^ would dissociate from **H** and precipitate in the form of NaH_2_PO_4_ salt. In such cases, no perturbations of chemicals shift proton signals would occur and the proton signals of Na**H**^+^ would disappear. Our experimental results clearly disagreed with the latter, as the Na**H**^+^ urea–proton signals were visible up to 4 eq. of added H_2_PO_4_^−^, featuring a drastic downfield shift (~3 ppm). On the other hand, the urea–proton signals ascribed to free **H** shifted towards the value measured for the **H**(H_2_PO_4_)_2_^2−^ complex (Figure S28a) [58]. These results unambiguously demonstrated that along with the precipitation of NaH_2_PO_4_ and the formation of **H**H_2_PO_4_^−^ and **H**(H_2_PO_4_)_2_^2−^ complexes, the formation of Na**H**H_2_PO_4_ and probably a complex including two dihydrogen phosphate ions (Na**H**(H_2_PO_4_)_2_^−^) also took place during the titration. The combination of processes exhibiting various exchange dynamics, along with the precipitation, prevented a detailed quantitative characterization of the underlying equilibria. However, the speciation (Figure S28c) calculated by assuming extensive Na^+^-induced cooperativity (Table S10) showcased that even under such conditions the precipitation of NaH_2_PO_4_ would be dominant, causing the disappearance of the Na**H**^+^ signals at the equivalence point (red region in Figure S28b). We could, therefore, conclude that (1) the precipitation of NaH_2_PO_4_ is a slow process, (2) the Na^+^-induced cooperativity in the binding of H_2_PO_4_^−^ by **H** is significantly more pronounced than the one observed for Cl^−^ and HSO_4_^−^, or (3) both.

## 3. Experimental Section

### 3.1. Materials

The synthetic procedure for the urea derivative of calixarene **H** is available in our recent publication [57]. The solvents, acetonitrile (MeCN; J.T. Baker (Phillipsburg, NJ, USA), HPLC-grade, ≤0.05% water; Fluka (Buchs, Switzerland), HPLC-grade, 0.01% water) and deuterated acetonitrile (Eurisotop (Saclay, France), +0.03% TMS, 99.80% D, <0.05% water), were used without further purification. For the physicochemical measurements, the following substances were used: NaCl (Carlo Erba (Cornaredo, Milan, Italy), p.a.), TEACl (Sigma-Aldrich (St. Louis, MO, USA), ≥98.0%), NaClO_4_ (Fluka, ≥98%), TBAClO_4_ (Sigma-Aldrich, ≥99.0%), TBAHSO_4_ (Sigma-Aldrich, ≥99.0%), TBAH_2_PO_4_ (Sigma-Aldrich, ≥99.0%), *tris*(hydroxymethyl)aminomethane (Merck (Darmstadt, Germany), EMPROVE^®^ EXPERT PhEur, BP, USP), HNO_3_ (aq, 2 mol dm^−3^), KCl (Gram-Mol (Zagreb, Croatia), p.a.), NaHSO_4_ (Kemika (Zagreb, Croatia), >99%,), NaH_2_PO_4_ × H_2_O (Kemika, p.a.).

### 3.2. Complexation of Alkali Metal Cations with Host Calixarene in Acetonitrile

Microcalorimetric measurements were performed using isothermal titration calorimeters from Microcal VP-ITC (Malvern Panalytical, Malvern, UK; *V*_cell_ = 1.43 mL) at 25.0 (**1**) °C. The enthalpy changes were recorded upon the stepwise, automatic addition of the titrant, i.e., a solution containing alkali metal cations (*c* = 1.3 × 10^−4^ to 1.5 × 10^−3^ mol dm^−3^, depending on the investigated system) to the titrand, i.e., a solution of calixarene **H** (*c* = 1 to 2 × 10^−4^ mol dm^−3^). Blank experiments were carried out to make corrections for the enthalpy changes corresponding to the dilution of the titrant solution in the pure solvent (MeCN). The dependence of the successive enthalpy change on the titrant volume was processed using Microcal OriginPro 7.0.

### 3.3. Solubility and Ion Pairing of Sodium Salts in Acetonitrile

#### 3.3.1. Sodium Chloride

##### Potentiometry—Method A

The solubility of NaCl was experimentally determined in the following way. First, saturated solutions of NaCl in pure acetonitrile and in TEACl–acetonitrile solutions (0.04–1 mmol dm^−3^; 100 mL in glass flask) were prepared by mixing (with a magnetic stirrer) an excess of solid NaCl in the solvent or solutions for at least 24 h. MeCN was then evaporated from the filtrated (PVDF ACRODISC LC [Pall Corporation, Port Washington, NY, USA] 0.2 μm) and saturated solution of NaCl of a known volume (90–99 mL). The resulting residue was dissolved in the small aliquot of Tris/TrisHNO_3_ buffer (pH = 9, *V* = 5 mL) and the pNa value of that solution was determined potentiometrically using a freshly calibrated (Figure S7) glass ion-selective electrode (ISE) for Na^+^ (Metrohm [Herisau, Switzerland] 6.0501.100). Prior to calibration, the ISE for Na^+^ was stored in 1 mol dm^−3^ NaCl (aq). As a reference cell, a Ag/AgCl electrode was used, filled with KCl (aq). The concentration of the inner filling solution was 3 mol dm^−3^, while for the outer filling a solution of 1 mol dm^−3^ KCl was utilized. Both electrodes were plugged in a Metrohm 913 pH Meter.

The reproducibility of the solubility results and the confidence of the Na-ISE readings were tested by using three individual saturated solutions of NaCl (only in the case without TEACl) and with the application of the internal standard method. In the latter case, the solution of NaCl (0.0512 mol dm^−3^) in Tris/TrisHNO_3_ buffer (aq, pH = 9) was added into the sample solution in cumulative volumes of 10 μL, 45 μL, and 155 μL, each causing a successive pNa change of ca 0.5.

When using the Excel Solver tool, the optimization criterion was the minimization of the sum of squared differences between the calculated and experimental values of the solubility of NaCl. The search area was reduced by setting constraints on the optimized variables using experimental findings and chemical logic. The used optimization algorithm was “standard LSGRG nonlinear” with default settings: precision = 10^−20^, convergence = 10^−4^, estimates = tangent, search = Newton. To circumvent the problem in the calculation with small numbers, the variable *K*_s_ and the object of optimization were multiplied by 10^9^ and 10^10^, respectively.

##### Potentiometry-Turbidimetry—Method B

Method B involved simultaneous potentiometric and turbidimetric titrations of NaClO_4_ (1 × 10^−4^ and 5 × 10^−4^ mol dm^−3^, *V*_0_ = 25 mL) with TEACl (1 × 10^−2^ mol dm^−3^) in acetonitrile with the ion strength being kept constant using TBAClO_4_ as an inert electrolyte (1 × 10^−2^ mol dm^−3^) for the preparation of the NaClO_4_ solution.

For measurements of pNa, the Na-ISE (Metrohm, 6.0501.100) was used in combination with the Ag/AgCl reference electrode (Metrohm, 6.0729.100) filled with TEACl (0.01 mol dm^−3^, CH_3_CN), both in the inner and outer filling spaces, and conditioned for 24 h in a solution identical to the electrode filling solution. Both electrodes were plugged in a Metrohm 913 pH Meter. Before each titration, the Na-ISE was freshly calibrated using NaCl solutions of known concentrations (Figure S8). In some titrations of NaClO_4_ with TEACl where pNa was measured, the titrant was added using an automated Hamilton titrator (250 μL), while in the others manual additions of the titrant were performed using Hamilton syringes (10–50 μL).

The precipitation of NaCl during the titration of NaClO_4_ with TEACl was followed by measuring the turbidity of the samples. For this purpose, a fiber optic probe (Cary 60, Agilent Technologies [Santa Clara, CA, USA]) was immersed in the thermostated (25.0 (**1**) °C) titration cell, and the recording parameters were set to Δ*λ* = 5 nm, average time = 0.2 s, and gap time = 0.5 to 2 min (depending on the frequency of the titrant addition).

The optimization criterion in method B was the minimization of the sum of squared differences between the calculated and experimental values of pNa. The settings for the Excel (Version 2504 Build 16.0.18730.20186) Solver tool were identical to those specified for method A. To circumvent the problem in the calculation with small numbers, variables *s* and *K*_s_ and the object of optimization were multiplied by 10^5^, 10^9^, and 10^4^, respectively.

### 3.4. Sodium Hydrogen Sulfate

#### 3.4.1. Flame AES

A saturated solution of NaHSO_4_ in MeCN was prepared by adding an excess of solid NaHSO_4_ (dried for 4 h at 115 °C prior to usage to remove water) in 2 Eppendorf tubes, each filled with 1.75 mL of MeCN, with the tubes being shaken for 3 days (on a Hettich Benelux shaker). The resulting suspension was then filtrated (PVDF ACRODISC LC, 0.2 μm). An aliquot of the filtrated merged solutions (3.00 mL) was transferred into a small (10 mL) glass beaker, which was placed in a heating oven for 1 h at 115 °C to evaporate the MeCN. The residue in the beaker was then dissolved (via sonification) in 5.00 g of ultrapure water (Milli-Q, MilliporeSigma, Burlington, MA, USA) and the solution was analyzed using a flame photometer (Buck Scientific Inc. [East Norwalk, Connecticut, USA] PFP-7). The photometer was calibrated with sodium standard solutions over the range of 1–10 ppm. The prepared solution of NaHSO_4_ was diluted by 10 times to enter the calibration range (final result = 1.6 ppm).

#### 3.4.2. Conductometry—Method C

The conductivity during the titration of NaClO_4_ with TBAHSO_4_ (concentrations given in Figure S10) was measured with a MettlerToledo (Greifensee, Switzerland) InLab 741-ISM conductivity cell (*K*_cell_ = 0.09806 cm^−1^) calibrated with a standard KCl solution (Merck, *κ* = 84.00 mS cm^−1^, *θ* = 25 °C) connected to a MettlerToledo SevenExcellence measuring device. The conductivity data were collected automatically (every 10 s) via MettlerToledo EasyDirect. The titrant solution (TBAHSO_4_) was added every 10 min in portions of 240 μL using Hamilton (Bonaduz, Switzerland) Autodilutor Microlab 500 equipped with a Hamilton syringe with a 250 μL volume and the appropriate ML 500 program. The temperature of the sample was kept constant at 25.0(1) °C using a JULABO GmbH (Seelbach, Germany) thermostat.

#### 3.4.3. ITC—Method D

The same experimental setup was used as in the investigation of the complexation of alkali metal cations with **H** (see above). The effect of small changes in ionic strength on the activity coefficients was neglected in methods C and D.

### 3.5. Sodium Dihydrogen Phosphate

#### 3.5.1. Potentiometry—Method E

The same procedure as for method A was used here. The NaH_2_PO_4_ × H_2_O was dried prior to usage for 4 h at 180 °C in order to remove water.

#### 3.5.2. Potentiometry–Turbidimetry—Method F

The same procedure as for method B was used here. The only differences were as follows: (1) titrant = TBAH_2_PO_4_; (2) for the concentrations of NaClO_4_ solutions, beside those used in method B, 1 mmol dm^−3^ was also used.

### 3.6. Cooperativity in Ion Pair Binding Measurements

#### 3.6.1. ITC

The ITC measurements were performed using similar experimental conditions to those used in the investigation of the complexation of alkali metal cations at **H**. The titrand was a solution of **H** with NaClO_4_ (*n*/*n* = 1, *c* = 0.2 mmol dm^−3^) in MeCN, whereas solutions of TEACl and TBAH_2_PO_4_ (both of *c* = 3.8 mmol dm^−3^) were used as titrants.

#### 3.6.2. UV

The UV spectrophotometric titrations were carried out at 25.0 ± 0.1 °C using an Agilent Cary 5000 spectrophotometer equipped with a thermostat. The spectral changes of the titrand solution of **H** with NaClO_4_ (*n*/*n* = 1) in MeCN (*c* ≈ 0.2 mmol dm^−3^; *V*_0_ = 2.2 mL) were recorded upon the stepwise addition of a titrant solution of TEACl (5 mmol dm^−3^) or TBAHSO_4_ (0.1 mol dm^−3^) into the measuring quartz cell (Hellma GmbH & Co. KG [Müllheim, Germany], Suprasil QX, *l* = 1 cm). The absorbances were sampled at 1 nm intervals, with an integration time of 0.2 s. The obtained spectrophotometric data were processed using the HypSpec (v. 1.01.0050) program [86]. 

#### 3.6.3. NMR

The ^1^H NMR spectra were recorded using Bruker (Billerica, MA, USA) Avance III HD 400 MHz/54 mm and Bruker Avance Neo 600 MHz/54 mm NMR spectrometers, equipped with an inverse broadband room temperature probe (5 mm PA BBI 1H/D–BB) and inverse triple-resonance TCl Prodigy cryoprobe (5 mm CPP1.1 TCl 600S3 H&F-CIN-D-05 XT), respectively. All proton spectra were acquired at 25.0 °C by using 64 K data points, a spectral width of 20 ppm, a recycle delay of 1.0 s, and 16 or 32 scans. CD_3_CN was used as a solvent and TMS as an internal standard for the proton chemical shifts. The ^1^H NMR titrations were performed by recording the spectral changes of the titrand solution composed of **H** and NaClO_4_ (*n*/*n* = 1, *c*_0_ = 0.2 to 0.9 mmol dm^−3^ depending on the identity of the titrant, *V*_0_ ≈ 0.5 mL) upon stepwise additions of the titrant solution, namely TEACl (7 mmol dm^−3^), TBAHSO_4_ (0.37 mol dm^−3^), or TBAH_2_PO_4_ (15 mmol dm^−3^). The dependences of the selected proton chemical shifts on the concentrations of the reactants were processed using the HYPNMR2008 program [87], whereas for the presentation of the results MestReNova (v. 14.2.0-26256) was used.

The data obtained using all methods were processed using Origin 7.5.

### 3.7. Molecular Dynamics

The molecular dynamics simulations were carried out using the GROMACS [88,89,90,91,92,93,94] package (version 2022.5). Intramolecular and nonbonded intermolecular interactions were modeled using the Charmm36 force field [95]. The initial structure of the free calixarene adopted a basket conformation of a flattened cone, whereas the initial structures of the calixarene complexes were constructed by placing the sodium cation in the center of lower-rim cavity between the ether oxygen atoms and the chloride anion between the urea groups of the lower-rim substituents. The calixarene and its complexes with Na^+^, Cl^−^, or both were solvated in cubical boxes (*a* = 6.5 nm) containing 3181–3183 acetonitrile molecules using the periodic boundary conditions. The solute concentration in such a box was 6 × 10^−3^ mol dm^−3^. The solvent boxes were equilibrated prior to the inclusion of calixarene (ion-complex), with the box density after equilibration in all cases being close to the experimental one (within 2%). The box was not neutralized during the simulations of the systems comprising calixarene and Na^+^ or Cl^−^. The calixarene (ion complex) was initially positioned in the center of the box. In all simulations, energy minimization, *NVT* equilibration (298.15 K, duration = 100 ps, time step = 1 fs, V-rescale algorithm [96], time constant = 0.1 ps), and *NpT* equilibration (1 bar, duration = 200 ps, time step = 1 fs, C-rescale algorithm [97], time constant = 2 ps) procedures were performed, followed by a molecular dynamics simulation in *NpT* conditions for 50 ns (260 ns). The Verlet algorithm [98] was employed with a time step of 1 fs. The cutoff radius for nonbonded van der Waals and short-range Coulomb interactions was 1.5 nm. Long-range Coulomb interactions were treated using the Ewald method as implemented in the PME (Particle Mesh Ewald) procedure [99]. The simulation temperature and pressure were kept constant during the simulation using the values and algorithms stated above. Data regarding the structure and energy were collected every 1 ps (10 ps). Figures of the structure of calixarene and its ion complexes were created using VMD (v. 1.9.3) software [100].

The criteria for defining coordination were as follows: (a) coordinating oxygen atoms for Na^+^ were identified by the conditions *d*(O−Na^+^) < 3 Å and 0° < ∠(C−O−Na^+^) < 180°; (b) coordinating NH groups for Cl^−^ were defined by *d*(NH−Cl^−^) < 2.9 Å and 90° < ∠(N−H−Cl^−^) < 180°; (c) intramolecular hydrogen bonds were characterized by *d*(NH−O) < 3.2 Å and 90° < ∠(N−H−O) < 180° [72,101]. The distribution of the coordination distances and angles for Na^+^ and Cl^−^, obtained from the MD simulations for Na**H**^+^ and **H**Cl^−^, are shown in Figure S18. Representative molecular structures of the most populated clusters of free calixarene and its ion complexes, classified by the solvent inclusion and coordination pattern, were determined using a principal component analysis (PCA) on a coordination matrix. The coordination matrix included the following: (1) the distances between the amide oxygens and NH groups of both urea moieties, as well as the distances between the urea oxygens and NH groups from both urea moieties (for **H**, Na**H**^+^, **H**Cl^−^, and Na**H**Cl); (2) the distances between Cl^−^ and the NH groups of both urea moieties (for **H**Cl^−^ and Na**H**Cl); (3) the distances between Na^+^ and ether, amide, and urea oxygens (for Na**H**^+^ and Na**H**Cl). For each distance specified above, the corresponding angles (anion/cation/oxygen—NH/CO/NH—NH/CO/NH) were also included in the coordination matrix. The structures closest to the centroids of the most populated clusters in the space defined by the first three principal components were selected as representative structures.

## 4. Conclusions

The heteroditopic *bis*(amide)-*bis*(urea) calix[4]arene host (**H**) exhibited high affinity for Na^+^ in MeCN, as determined via ITC. The sodium-induced cooperativity in the binding of several anions with moderate affinity for **H** (Cl⁻, HSO_4_⁻, H_2_PO_4_⁻) in MeCN was subsequently investigated using a combination of several techniques (NMR, ITC, and UV). To achieve a comprehensive thermodynamic understanding of the equilibria in solution, ion pairing phenomena and the precipitation of the investigated salts were characterized. The strength of the ion pairing followed the trend of NaCl < NaHSO_4_ < NaH_2_PO_4_, whereas the solubility exhibited a different sequence of NaH_2_PO_4_ < NaCl < NaHSO_4_. Although the experiments indicated that **H** binds the NaH_2_PO_4_ ion pair, the extremely low solubility and favorable ion pairing precluded the quantitative evaluation of the related cooperativity. In contrast, for both NaCl and NaHSO_4_, significant positive cation-induced cooperativity (approximately one order of magnitude increase in complex stability constants) was observed. The cooperativity was quantified using models of varying levels of complexity. The results obtained by the simple model (commonly employed in reported studies) were comparable to those based on a more elaborate thermodynamic model. Higher cooperativity was observed for NaCl compared to NaHSO_4_. The MD simulations revealed that the conformations of the ternary complex comprising Na^+^, Cl^−^, and **H** include a host-separated (predominant) and contact ion pair. The structural analysis of the MD data suggested that the observed positive cooperativity for Na**H**Cl formation is caused by Coulombic interactions between the bound ions, favorable rearrangements of intramolecular hydrogen bonds, and the inclusion of an acetonitrile molecule (absent in the **H**Cl^−^ complex). Overall, this work showcased that the reliable thermodynamic characterization of ion pair complex formation demands the consideration of several equilibria and presented the details of a multimethod experimental approach of dealing with this task. Applying this comprehensive approach could guide the design of selective electrochemical sensors optimized for the analysis of nonaqueous industrial effluents, where accounting for ion pairing and solubility ensures accurate signal interpretation under varying conditions. We hope this work will encourage researchers in the field to adopt such thorough methodologies in future studies, enhancing the development of tailored supramolecular systems for practical applications.

## Figures and Tables

**Table 1:** Thermodynamic parameters for complexation of alkali metal cations with calixarene **H** (1:1 complex stoichiometry) in MeCN at 25 °C, determined via ITC ^a^.

Cation:	Li^+^	Na^+^	K^+^
log *K*_C**H**_	≥7	6.69(1)	2.80(1)
Δ_r_*G°*/kJ mol^−1^	≤−43	−38(1)	−15.9(4)
Δ_r_*H°*/kJ mol^−1^	−36.4(1)	−47.6(9)	−36.8(3)
(−*T*·Δ_r_*S*°)/kJ mol^−1^	≤−7	9.2(9)	20.9(3)

^a^ Uncertainties of the last digit are given in parentheses as standard errors of the mean (*N* = 3 or 4).

**Table 2:** Solubility (*s*), solubility product (*K*_s_), and ion pairing constant (*K*_IP_) values for NaCl in acetonitrile at 25 °C obtained using two methods (A and B) described in the text.

	Method A	Method B	Average ^a^
10^5^ *s*/mol dm^−3^	4.8(3) ^a^	7.4(2) ^b,c^	6(1)
−log (*s*/mol dm^−3^)	4.32(3) ^a^	4.13(1) ^b^	4.22(9)
−log (*K*_s_/mol^2^ dm^−6^)	8.77(4) ^b^	8.41(3) ^a^	8.6(2)
log (*K*_IP_/mol^−1^ dm^3^)	3.5(1) ^b^	3.5(1) ^a^	3.4(1)

^a^ Uncertainty of the last digit is given in parentheses as standard error of the mean (*N* = 2 or 3). ^b^ Uncertainty of the last digit is given in parentheses as standard error calculated from the covariance matrix σ^2^∙(J^τ^J)^−1^ using the Jacobian at best-fit values. ^c^ Fitting result from one titration curve; fixed value for other titration curves.

**Table 3:** Solubility (*s*), solubility product (*K*_s_), and ion pairing constant (*K*_IP_) values for NaHSO_4_ in acetonitrile at 25 °C.

Method	Flame AES		
10^3^ *s*/mol dm^−3^	1.2(1) ^b^		
Method	C: conductometry	D: ITC	average
log (*K*_IP_/mol^−1^ dm^3^)	4.22(2) ^a^	4.60 (6) ^b,c^	4.4(2)
			average
−log (*K*_s_/mol^2^ dm^−6^)	7.24(6) ^d^	7.58(7) ^e^	7.4(2)

^a^ Uncertainty of the last digit is given in parentheses as standard deviation from the fitting program. ^b^ Uncertainty of the last digit is given in parentheses as standard error of the mean (*N* = 3). ^c^ Other calculated thermodynamic data describing ion pairing of NaHSO_4_ are Δ_r_*H*° = −7.2(2) kJ mol^−1^, −*T*·Δ_r_*S*° = −19.1(3) kJ mol^−1^. ^d^ Calculated using Equation (6) and data obtained via flame AES and conductometry (method C). ^e^ Calculated using Equation (6) and data obtained via flame AES and ITC (method D).

**Table 4:** Solubility (*s*), solubility product (*K*_s_), and ion pairing constant (*K*_IP_) values for NaH_2_PO_4_ in acetonitrile at 25 °C obtained using two methods.

	Method E	Method F
10^6^ *s*/mol dm^−3^	2.7(3) ^a,b^	2.7 ^b^
−log (*K*_s_/mol^2^ dm^−6^)	13.6(4) ^a^	13.7(1) ^a^
log (*K*_IP_/mol^−1^ dm^3^)	8.6(4) ^a^	8.1(1) ^a^

^a^ Uncertainty of the last digit is given in parentheses as standard error of the mean (*N* = 2 or 3). ^b^ The value was held constant during the optimization procedure for both the *K*_s_ and *K*_IP_ values.

**Table 5:** Stability constants for anion (A) binding to the sodium complex of calixarene **H** in MeCN at 25 °C and the extent of cation-induced cooperativity (Δlog *K*) ^a^.

A	Method	log *K*_HA_	log *K*_NaHA_	Δlog *K*
Cl^−^	NMR UV ITC	2.06(1) ^b^ 2.22(3) ^b^ 2.47(6) ^b^	3.27(2) ^c^ 3.38(1) ^c^, 3.32 (1) ^d^, 3.31 (1) ^e^ 3.40(2) ^c^	1.21 1.09 0.93
HSO_4_^–^	NMR UV	1.72(2) ^b^ 1.74(3) ^b^	2.45(1) ^c^ 2.33(4) ^c^, 2.29 (9) ^d^, 2.40 (1) ^e^	0.73 0.66

^a^ Uncertainties of the last digit are given in parentheses as standard errors of the mean (*N* = 3–5) or standard deviations in the case of NMR titrations. ^b^ Reported in our previous work [58]. ^c^ Calculated using the simple model—with the assumption that all **H** was in the form of inseparable Na**H**^+^ at the beginning of titration and with neglecting ion pairing in the solution (Model 1). ^d^ Calculated using the model that included the complexation constants for the formation of: Na**H**^+^(sln), **H**A^−^(sln), and Na**H**A(sln) but ignored the ion pairing (formation of NaA(sln)) (Model 2). ^e^ Calculated using the complete model, which included the complexation constants for the formation of: Na**H**^+^(sln), **H**A^−^(sln), Na**H**A(sln), and NaA(sln) (Model 3). The value of the ion pairing constant for NaCl was taken as the average of the values obtained by methods A and B (described in the previous section), whereas in the case of NaHSO_4_, it was possible to determine the value of *K*_IP_ through the refinement procedure (along with the value of *β*(Na**H**HSO_4_)), with the result (log *K*_IP_ = 4.3 (**1**)) being in good agreement with the ones gained by methods C and D (log *K*_IP_ = 4.4).

**Table 6:** Thermodynamic parameters for binding of chloride by sodium complex with **H** determined via ITC in acetonitrile at 25 °C ^b,c,d^.

	log *K*	Δ_r_*H°*/kJ mol^−1^	−*T*·Δ_r_*S*°/kJ mol^−1^
Na**H**^+^ + Cl^−^	3.40(2) ^a^	−18.2(7)	−1.2(6)

^a^ Uncertainties of the last digit are given in parentheses as standard errors of the mean (*N* = 3 or 4). ^b^ For comparison, the values of log *K*, Δ_r_*H°*/kJ mol^−1^, and −*T*·Δ_r_*S°*/kJ mol^−1^ for the complexation **H** + Cl^−^ in MeCN characterized in the work of Cvetnić et al. [58] were 2.47, −5, and −8.9, respectively. ^c^ The dilution heats of TBAHSO_4_ were too big for reliable calculation of the thermodynamic parameters. ^d^ The solubility product of NaH_2_PO_4_ was too low to enable the experimental evaluation of these parameters.

**Table 7:** The changes in several MD-obtained parameters between the initial and final structures of the complex, comparing the complexation of Cl^−^ with free **H** vs. complexation with Na**H**^+^ in MeCN (50 ns, step = 1 ps, 298.15 K, 1 bar). The values of the parameters are expressed as average numbers.

	Coord. Number for Na^+^	Coord. Number for Cl^−^	Number of Intramolecular Hydrogen Bonds	% Structures with Included Solvent Molecule
**H**	−	−	0.9	63
**H**Cl^−^	−	4.0	0	2
Na**H**⁺	6.0	−	0.1	99
Na**H**Cl	5.4	2.6	0.3	97

## Data Availability

All data supporting the findings of this study are available in the Supplementary Materials.

## Supplementary Materials

The following supporting information can be downloaded at: https://www.mdpi.com/article/10.3390/molecules30112464/s1, Figure S1: ^1^H NMR spectra of mixtures of calixarene **H** with various relevant tetralkylammonium salts. Figure S2: ITC titration of **H** with LiClO_4_. Figure S3: UV titration of **H** with NaClO_4_. Figure S4: ITC titration of **H** with KClO_4_. Figure S5: Comparison of thermodynamic parameters for the complexation of **H** and related carbonyl calix[4]arene derivatives with alkali metal cations. Figure S6. Conductometric titration of NaClO_4_ with TEACl. Figure S7. Calibration of Na-ISE in Tris/TrisHNO_3_ buffer. Figure S8. Calibration of Na-ISE in TBAClO_4_ (MeCN). Figure S9. Potentiometric-turbidimetric titration of NaClO_4_ with TEACl in TBAClO_4_ (MeCN). Figure S10. Conductometric titration of NaClO_4_ with TBAHSO_4_. Table S1. Values of molar ionic conductivities regarding Figure S10. Figure S11. Conductivity measurement of TBAHSO_4_ solutions. Figure S12. Solubility of NaH_2_PO_4_ in acetonitrile at different concentrations of TBAH_2_PO_4_. Physico-chemical model used in Method E. Figure S13. The program used within Method E. Figure S14. Potentiometric-turbidimetric titration of NaClO_4_ with TBAH_2_PO_4_. Table S2. Model used for fitting potentiometric titration data depicted in Figure 5 and Figure S14. Physico-chemical model used in Method F. Table S3. Chemical shifts of proton signals calculated for calixarene **H** in the form of Na**H**Cl. Table S4. Model used for fitting the UV titration data depicted in Figure S15. Figure S15. UV titration of Na**H**ClO_4_ with TEACl. Figure S16. ITC titration of Na**H**ClO_4_ with TEACl. Figure S17. Distance between pairs of opposite upper rim phenyl carbons at **H** during MD simulation of free **H**. Figure S18. Histograms showing distributions of distances and angles relevant to the performed MD simulations with **H** and its complexes. Table S5. Time-averaged coordination numbers for Na^+^ and Cl^−^, and time-averaged numbers of intramolecular hydrogen bonds in **H**, **H**Cl^−^, Na**H**^+^, and Na**H**Cl, obtained by MD simulation. Table S6. Structural analysis of the results of MD simulations. Table S7. Representative clusters of structures obtained by MD simulation. Figure S19. Index number of acetonitrile molecules that occupy the hydrophobic cavity of **H** during MD simulations. Figure S20. (a) Distance and (b) potential energy between Na^+^ and Cl^−^ at **H** during MD simulation of Na**H**Cl (50 ns). Figure S21. The distribution analysis results for data in Figure S20. Figure S22. (a) Distance and (b) potential energy between Na^+^ and Cl^−^ at **H** during MD simulation of Na**H**Cl (260 ns). Figure S23. The distribution analysis results for the data in Figure S22. Figure S24. ^1^H NMR spectroscopy titration of Na**H**ClO_4_ with TBAHSO_4_. Table S8. Chemical shifts of proton signals calculated for calixarene **H** in the form of Na**H**HSO_4_. Table S9. Model used for fitting data depicted in Figure S26. Figure S25. Simulation of NMR titration depicted in Figure S24. Figure S26. UV titration of Na**H**ClO_4_ with TBAHSO_4_. Figure S27. ITC titration of Na**H**ClO_4_ with NaH_2_PO_4_. Figure S28. NMR titration of Na**H**ClO_4_ with NaH_2_PO_4_. Table S10. Model used for creating distribution depicted in Figure S28d.
