# Supplementary material for: A Detailed Thermodynamic Description of Ion Pair Binding by a Calix[4]arene Derivative Containing Urea and Amide Functionalities
Source: Molecules. 2025 Jun 4;30(11):2464. doi: 10.3390/molecules30112464 (PMC12157717; doi:10.3390/molecules30112464)
Supplement: Supplementary file 1 [file molecules-30-02464-s001.zip › molecules-3622391-supplementary.pdf]

A Detailed Thermodynamic Description of Ion Pair Binding by a Calix[4]arene Derivative Containing Urea and Amide Functionalities

Marija Cvetnić,[1] Tamara Rinkovec,[2] Robert Vianello,[2] Gordan Horvat,[1] Nikola Bregović*,[1] and Vladislav Tomišić*[1]

[1] Division of Physical Chemistry, Department of Chemistry, Faculty of Science, University of Zagreb, Horvatovac 102a, 10000 Zagreb, Croatia

[2] Laboratory for the Computational Design and Synthesis of Functional Materials, Division of Organic Chemistry and Biochemistry, Ruđer Bošković Institute, Bijenička cesta 54, 10000 Zagreb, Croatia

*Correspondence: nbregovic@chem.pmf.hr, vtomisic@chem.pmf.hr

SUPPORTING INFORMATION

Contents

1. Complexation of alkali metal cations with host calixarene in acetonitrile 3
2. Solubility and ion pairing of selected sodium salts in acetonitrile 6
Sodium chloride 6
Method A 6
Method B 7
Sodium hydrogen sulfate 8
Method C 8
Sodium dihydrogen phosphate 9
Method E 9
Method F 12
3. Cooperativity in binding of sodium ion pairs at host calixarene in acetonitrile 14
Sodium chloride 14
Molecular Dynamics 16
Sodium hydrogen sulfate 24
Sodium dihydrogen phosphate 27
4. Literature 29

1. Complexation of alkali metal cations with host calixarene in acetonitrile

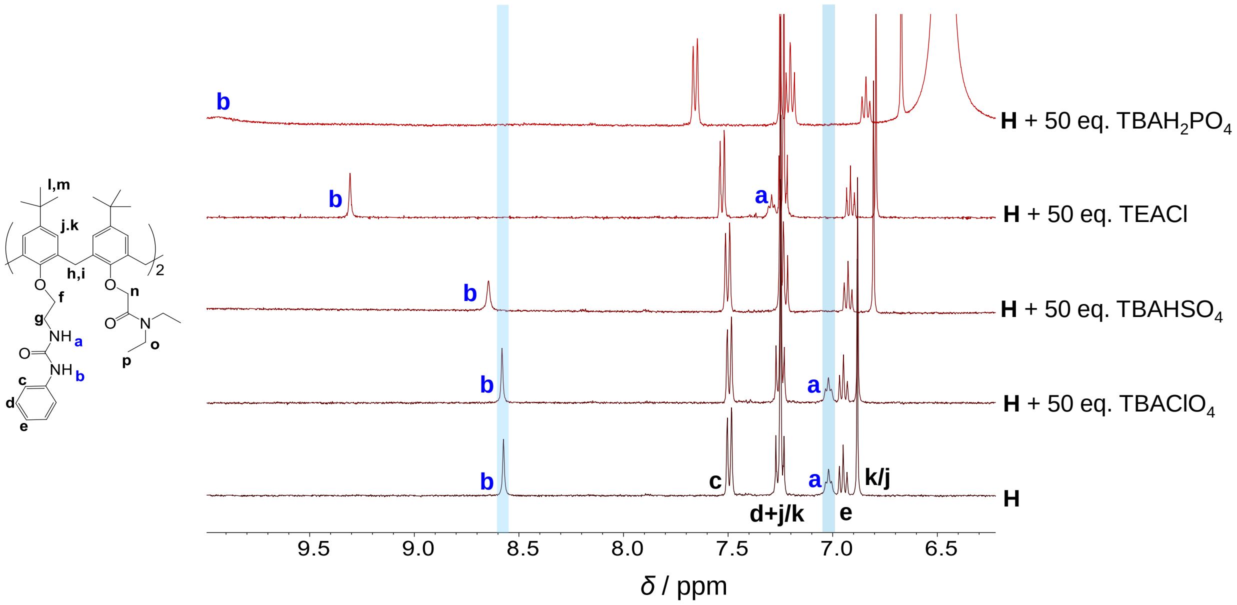

Figure S1. ^{1}H NMR spectra of mixtures of calixarene **H** with 50 molar equivalents (eq.) of various relevant tetralkylammonium salts in CD_3CN (400 MHz, 25 °C, c(**H**) = 1 mmol dm^{-3}). Chemical shifts for protons at **H** demonstrate that it does not bind ClO_4^-, unlike other tested anions (HSO_4^-, Cl^-, and $H_2PO_4^-$) where change of proton chemical shifts is obvious, especially for ureido-protons (**a** and **b**).

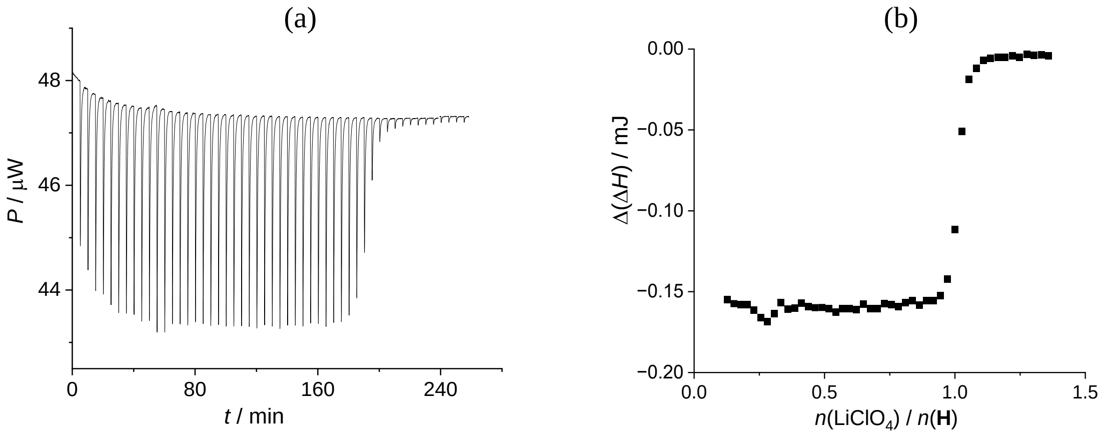

Figure S2. a) Microcalorimetric titration of **H** (c = 1.22×10^{-4} mol dm^{-3}, V_0 = 1.425 mL) with $LiClO_4$ (c = 1.466×10^{-3} mol dm^{-3}) in acetonitrile at 25 °C. b) Dependence of successive enthalpy change on $n(LiClO_4)$ / n(**H**) ratio.

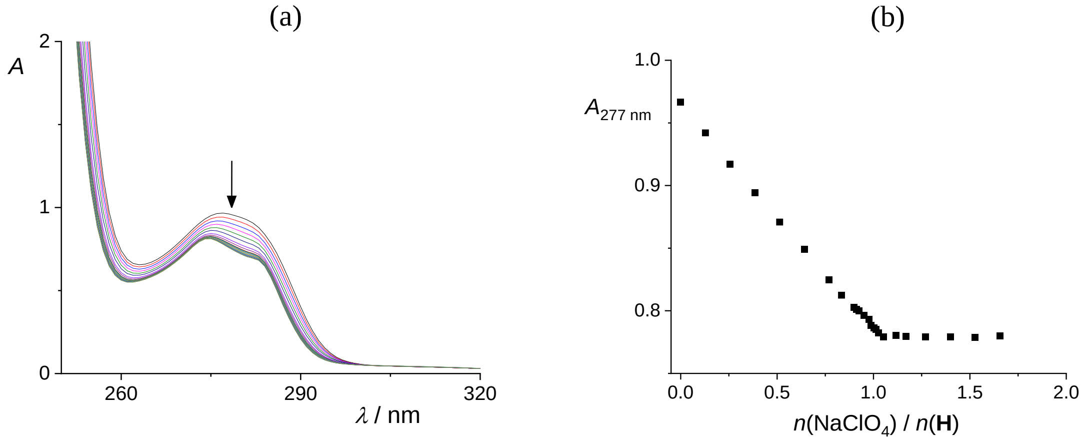

Figure S3. a) Spectrophotometric titration of **H** (c = 1.42×10^{-4} mol dm^{-3}, V_0 = 2.2 mL) with $NaClO_4$ (c = 8.00×10^{-4} mol dm^{-3}) in acetonitrile at 25 °C. l = 1 cm; ϑ = (25.0 ± 0.1) °C. The spectra are corrected for dilution. b) Dependence of absorbance at 277 nm on $n(NaClO_4)$ / n(**H**) ratio. ■ experimental; — calculated. *Experimental details: Agilent Cary 5000 spectrophotometer equipped with a thermostatting device and the measuring quartz cell Hellma, Suprasil QX were used. Absorbances were sampled at 1 nm intervals, with an integration time of 0.2 s.*

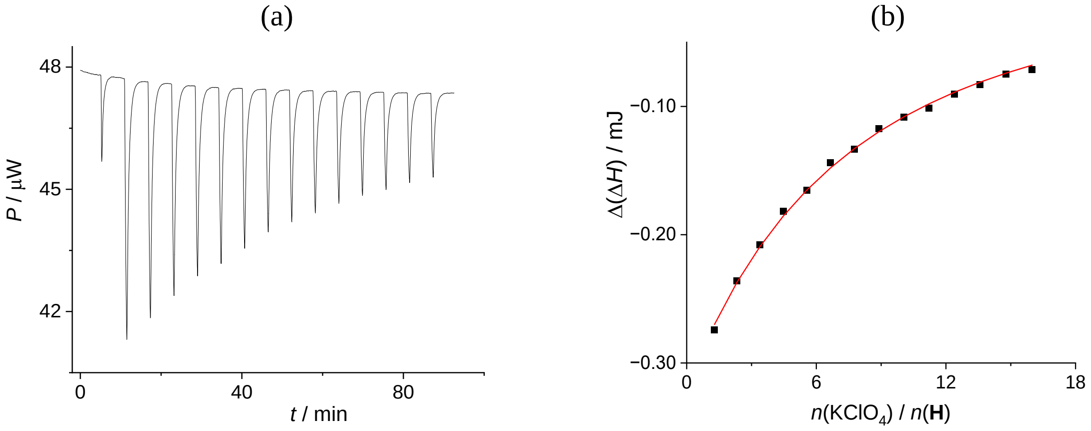

Figure S4. a) Microcalorimetric titration of **H** (c = 1.00×10^{-4} mol dm^{-3}, V_0 = 1.425 mL) with $KClO_4$ (c = 7.05×10^{-4} mol dm^{-3}) in acetonitrile at 25 °C. b) Dependence of successive enthalpy change on $n(KClO_4)$ / n(**H**) ratio. ■ experimental; — calculated.

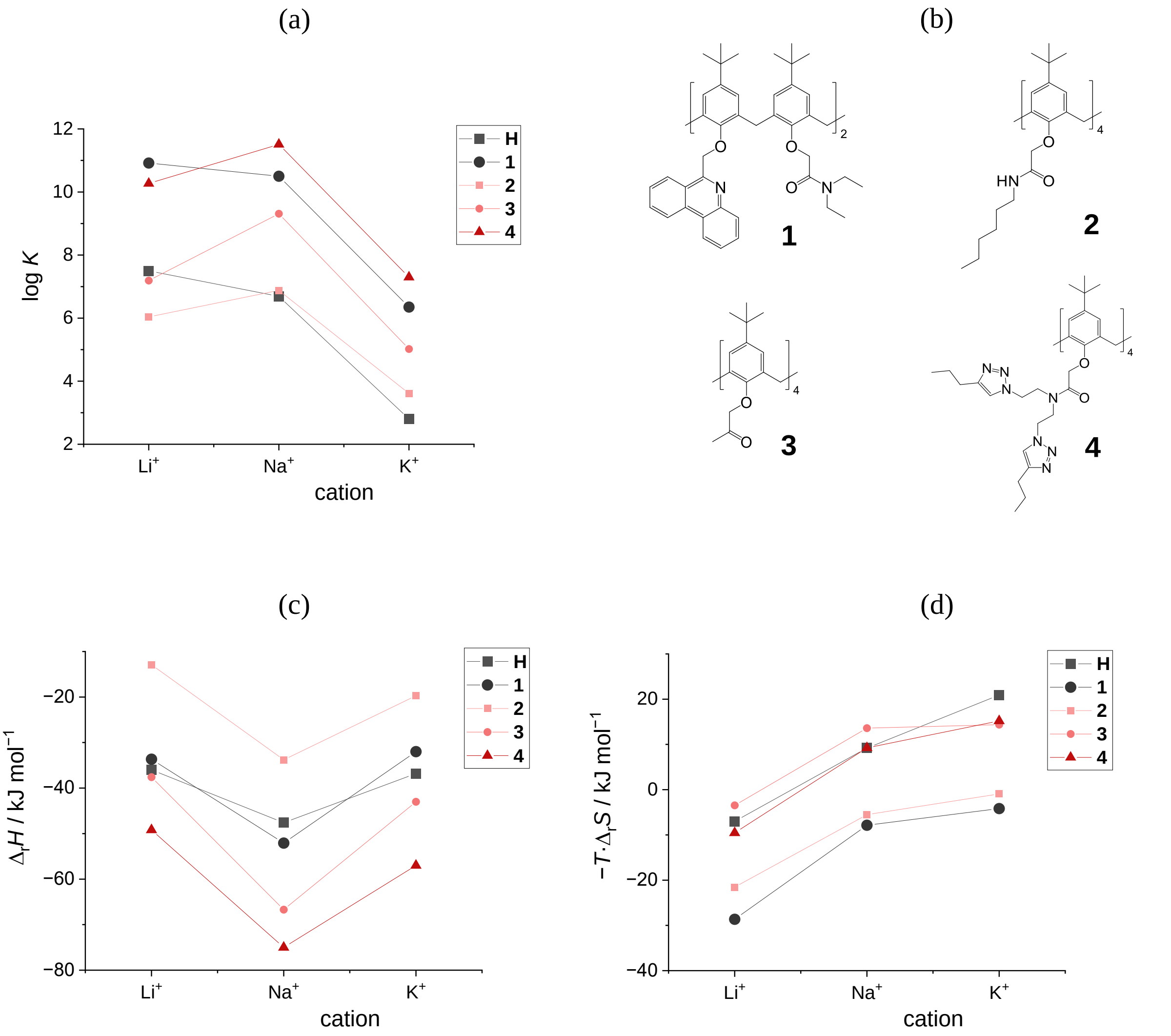

Figure S5. Comparison of thermodynamic parameters obtained for the complexation of the herein investigated amide-urea-calix[4]arene derivative **H** and several other lower-rim *bis*-(in black) or *tetra*-(in red) carbonyl calix[4]arene derivatives (**1**,[1] **2**,[2] **3**,[3] **4**[4]) with alkali metal cations in MeCN at 25 °C.

2. Solubility and ion pairing of selected sodium salts in acetonitrile

Sodium chloride

Prior to determination of K_{sp}, K_{IP} and s for NaCl in MeCN, conductometric titration was performed (Figure S6). However, due to relatively high concentrations of the reactants used, in comparison with the values of K_{sp}, K_{IP} and s (later obtained), it was concluded that the precipitation of NaCl was probably happening along with ion pairing so these data could not be used for K_{IP} evaluation.

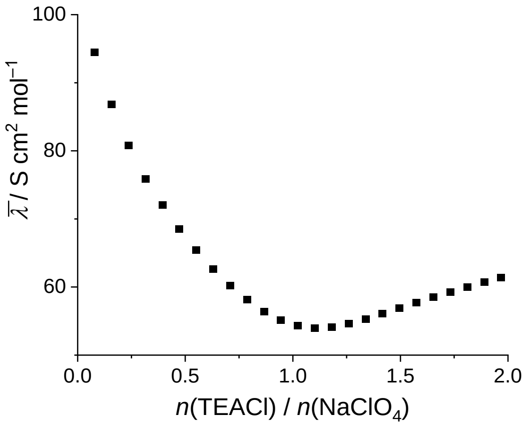

Figure S6. Conductometric titration of $NaClO_4$ (c = 6.57×10^{-4} mol dm^{-3}, V_0 = 30.0 mL) with TEACl (c = 5.03×10^{-3} mol dm^{-3}) in acetonitrile at (25.0 ± 0.1) °C. Molar ionic conductivity was averaged using the sum of concentrations for all ions at every point of titration.

Method A

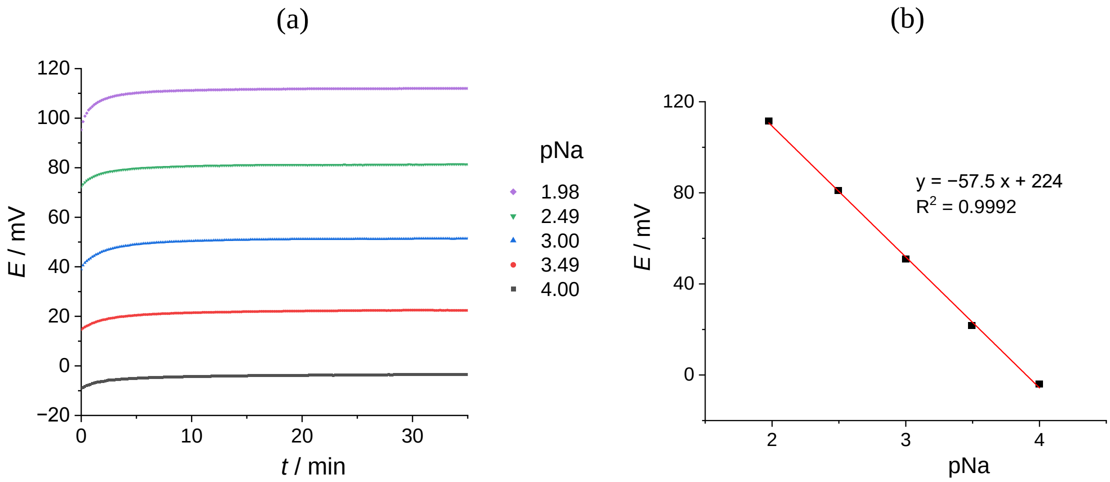

Figure S7. Calibration of glass ion-selective electrode for Na^+ in a series of calibration solutions of NaCl in Tris/TrisHNO_3 buffer (pH = 9) at 25 °C. Average potential in the range 13 – 15 min (a) was used for drawing calibration curve (b).

Method B

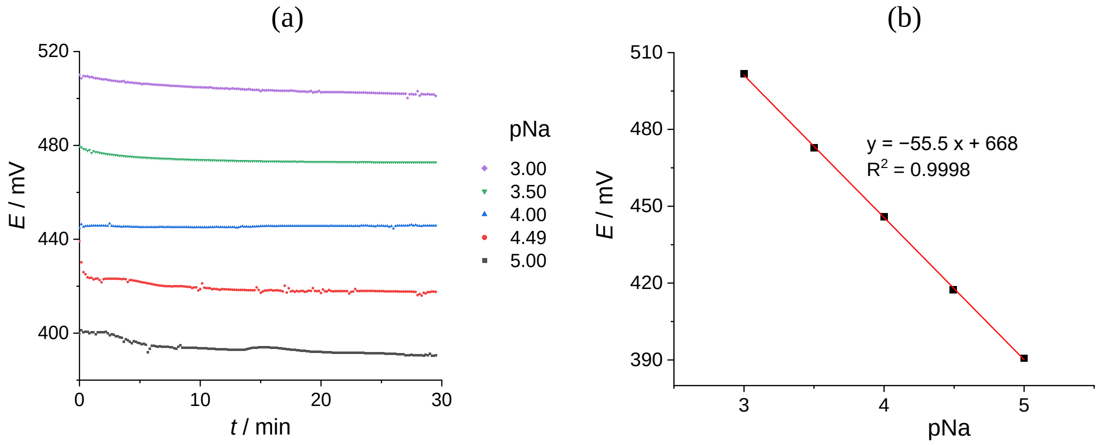

Figure S8. Calibration of glass ion-selective electrode for Na^+ in a series of calibration solutions of $NaClO_4$ (1.00×10^{-5} to 1.00×10^{-3} mol dm^{-3}) in $TBAClO_4$ (1.00×10^{-2} mol dm^{-3}, sln in CH_3CN) at 25 °C. Average potential in the range 28.5 – 29.5 min (a) was used for drawing calibration curve (b). The potential readings were collected either automatically (every 10 seconds, as shown here) or manually, with the criterion for stability being a variation of less than 0.3 mV/min.

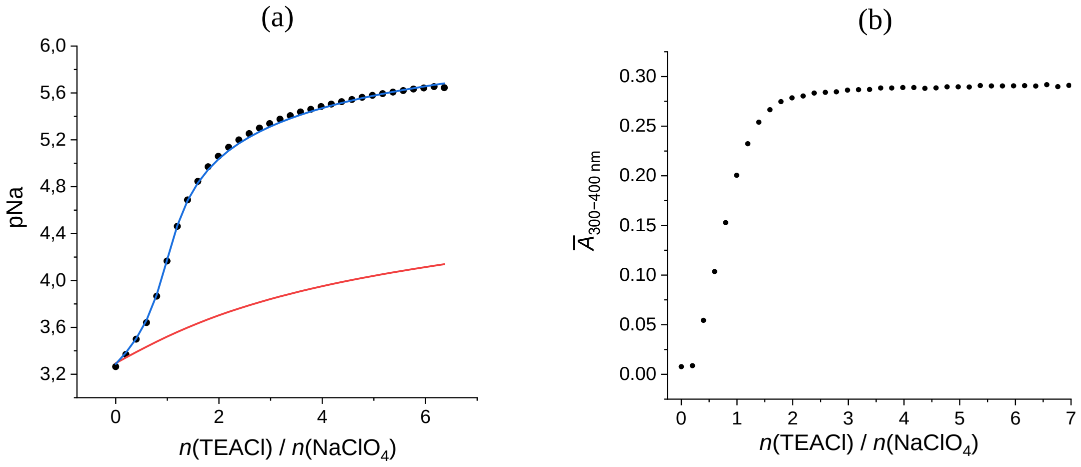

Figure S9. Potentiometric-turbidimetric titration of $NaClO_4$ (5.07×10^{-4} mol dm^{-3}) with TEACl (1.01×10^{-2} mol dm^{-3}) in acetonitrile solution of $TBAClO_4$ ($c = 1\times10^{-2}$ mol dm^{-3}, V = 25 mL). For the description of the experimental potentiometric data (●), two models were used: including NaCl precipitation (—); omitting NaCl precipitation (—).

Sodium hydrogen sulfate

Method C

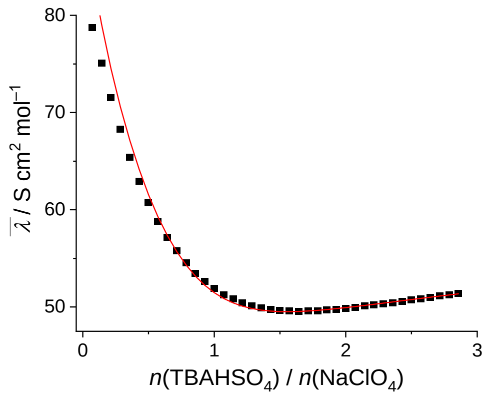

Figure S10. Conductometric titration of $NaClO_4$ (c = 5.08×10^{-4} mol dm^{-3}, V_0 = 30.0 mL) with $TBAHSO_4$ (c = 4.53×10^{-3} mol dm^{-3}) in acetonitrile at (25.0 ± 0.1) °C. ■ experimental; — calculated. Molar ionic conductivity was averaged using the sum of concentrations for all ions at every point of titration.

Table S1. Values of molar ionic conductivities used from the literature (lit)[5] or calculated from the fitting of titration curve obtained during the titration of $NaClO_4$ with $TBAHSO_4$ depicted in Figure S10 (calc).

ion	Na^+	ClO_4^-	TBA^+	HSO_4^-
λ_∞ / S cm^2 mol^{-1}	77^{lit}	103.6^{lit}	61.63^{lit}	57.3^{calc}

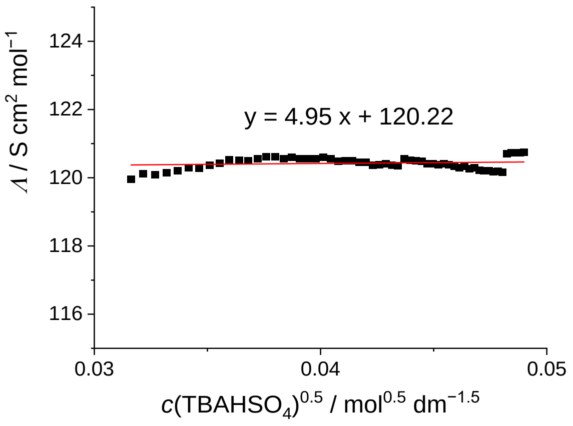

Figure S11. Conductivity measurement of $TBAHSO_4$ solutions (c = 8.3×10^{-4} − 2.4×10^{-3} mol dm^{-3}). ■ experimental; — calculated.

Sodium dihydrogen phosphate

Method E

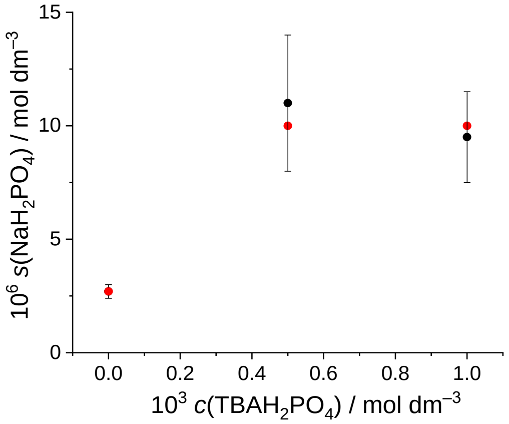

Figure S12. Solubility of NaH_2PO_4 in acetonitrile at different concentrations of $TBAH_2PO_4$ at 25 °C. ● experimental; ● calculated using method E.

Physico-chemical model used in Method E

Definition of chemical equilibria and the appropriate equilibrium concentration constants used in the evaluation of solubility product (K_{sp}) and ion-pairing constant (K_{IP}) for NaH_2PO_4 in acetonitrile are given in equations (S1 – S8):

$$\mathrm{NaH_2PO_4(s)} \rightleftarrows \mathrm{Na^+(sln)} + \mathrm{H_2PO_4^-(sln)} \quad \text{(S1)}$$

$$\mathrm{Na^+(sln)} + \mathrm{H_2PO_4^-(sln)} \rightleftarrows \mathrm{NaH_2PO_4(sln)} \quad \text{(S2)}$$

$$\mathrm{NaH_2PO_4(s)} \rightleftarrows \mathrm{NaH_2PO_4(sln)} \quad \text{(S3)}$$

$$2\ \mathrm{H_2PO_4^-(sln)} \rightleftarrows \mathrm{(H_2PO_4)_2^{2-}(sln)} \quad \text{(S4)}$$

$$K_{\mathrm{sp}}(\mathrm{NaH_2PO_4}) = [\mathrm{Na^+}]_{\mathrm{sat}} \cdot [\mathrm{H_2PO_4^-}]_{\mathrm{sat}} \quad \text{(S5)}$$

$$K_{\mathrm{IP}}(\mathrm{NaH_2PO_4}) = [\mathrm{NaH_2PO_4}] \,/\, [\mathrm{Na^+}]\cdot[\mathrm{H_2PO_4^-}] \quad \text{(S6)}$$

$$K_{\mathrm{SIP}}(\mathrm{NaH_2PO_4}) = [\mathrm{NaH_2PO_4}]_{\mathrm{sat}} = K_{\mathrm{IP}}(\mathrm{NaH_2PO_4}) \cdot K_{\mathrm{sp}}(\mathrm{NaH_2PO_4}) \quad \text{(S7)}$$

$$K_{\mathrm{dim}}((\mathrm{H_2PO_4})_2^{2-}) = [(\mathrm{H_2PO_4})_2^{2-}] \,/\, [\mathrm{H_2PO_4^-}]^2 \quad \text{(S8)}$$

The solubility of NaH_2PO_4 in acetonitrile is equal to the sum of the concentrations of Na^+ and dissolved NaH_2PO_4 in the saturated solution:

$$s(\mathrm{NaH_2PO_4}) = [\mathrm{Na^+}]_{\mathrm{sat}} + [\mathrm{NaH_2PO_4}]_{\mathrm{sat}} \quad \text{(S9)}$$

but it can also be written in terms of concentration of $H_2PO_4^-$ as:

$$s(\mathrm{NaH_2PO_4}) = [\mathrm{H_2PO_4^-}]_{\mathrm{sat}} + 2[(\mathrm{H_2PO_4})_2^{2-}]_{\mathrm{sat}} + [\mathrm{NaH_2PO_4}]_{\mathrm{sat}} \quad \text{(S10)}$$

When NaH_2PO_4 is dissolved in the solution of $TBAH_2PO_4$ in MeCN, the concentration of dissolved Na^+, i.e. the solubility of NaH_2PO_4 is given by the following equation:

$$s(\mathrm{NaH_2PO_4}) = [\mathrm{H_2PO_4^-}]_{\mathrm{sat}} + 2[(\mathrm{H_2PO_4})_2^{2-}]_{\mathrm{sat}} + [\mathrm{NaH_2PO_4}]_{\mathrm{sat}} - c(\mathrm{TBAH_2PO_4}) \quad \text{(S11)}$$

Using equations (S5 – S9 and S11), the solubility of NaH_2PO_4 (s) in the presence of $TBAH_2PO_4$ can be written as an implicit function of $c(TBAH_2PO_4)$ (= c), K_{sp}, K_{IP}, and K_{dim}:

$$s^3 + s^2 \cdot (c - 3K_{sp}K_{IP}) + s \cdot \left(3{K_{sp}}^2{K_{IP}}^2 - K_{sp} \cdot (1 + 2cK_{IP})\right) + \\ + {K_{sp}}^2 \cdot \left(K_{IP} - 2K_{dim} + c{K_{IP}}^2\right) - {K_{sp}}^3{K_{IP}}^3 = 0 \qquad (S12)$$

Applying initial reasonable estimations for the values of K_{sp} (10^{-15}) and K_{IP} (10^5) and the value of K_{dim} known from the literature,[6] the solubilities of NaCl were calculated using eq. (S12) for different concentrations of $TBAH_2PO_4$. By running our own optimization program (Figure S13) specifically for this problem, the optimized values of constants K_{sp} and K_{IP} were obtained. The optimization criterion was the minimization of the sum of squared differences between calculated and experimental values for the solubility of NaH_2PO_4 (obtained under different $c(TBAH_2PO_4)$). The search area was reduced by setting constraints on optimized variables using experimental findings and chemical logic. As the value of solubility of NaH_2PO_4 in acetonitrile without $TBAH_2PO_4$ was reproducibly obtained, it was used as a fixed parameter in the optimization procedure.

```python
from math import *

Kdim = 2398.83

def f(s, c, Kip, Ksi):
    A = 1
    B = c - (3.0 * Ksi * Kip)
    C = 3 * Ksi**2 * Kip**2 - (Ksi*(1 + 2 * c * Kip))
    D = Ksi**2 * (Kip - 2 * Kdim + c * Kip**2) - (Ksi**3 * Kip**3)
    res = A * (s**3) + B * (s**2) + C * s + D
    return res

def solver(c, Kip, Ksi):
    s = 0.0
    min = 10
    resS = 100
    while s < 0.00003:
        res = f(s, c, Kip, Ksi)
        if  abs(res) < abs(min):
            min = res
            resS = s
        s = s + 1.0e-7
    return resS

c1 = 0.0
c2 = 0.0005
c3 = 0.001

s1exp = 2.67e-06
s2exp = 1.07e-05
s3exp = 9.47e-06

min = 10

print("Ksi    | Kip    | s1    | s2    | s3    | odstupanje1 | odstupanje2 | odstupanje3 | sumaOdstupanja")
Ksi = 1.0e-15
Kip = 1.0e5

while Ksi < 1.0e-12:
    Kip = 1.0e5
    while Kip < 1.0e10:
        s1 = s1exp
        s2 = solver(c2, Kip, Ksi)
        s3 = solver(c3, Kip, Ksi)


        odstupanje1 = (s1 - s1exp)**2
        odstupanje2 = (s2 - s2exp)**2
        odstupanje3 = (s3 - s3exp)**2

        sumaOdstupanja = odstupanje1 + odstupanje2 + odstupanje3

        if min > sumaOdstupanja:
            min = sumaOdstupanja
            print(str(Ksi) + " ; " + str(Kip) + " ; " + str(s1) + " ; " + str(s2) + " ; " + str(s3) + " ; " + str(odstupanje1) +

        Kip = Kip * 1.1
    Ksi = Ksi + 1e-15

print ("Kraj")
```

Figure S13. The program developed to calculate K_{sp} (denoted as “Ksi”) and K_{IP} (denoted as “Kip”) for NaH_2PO_4 in acetonitrile using experimental data pairs (solubility of NaH_2PO_4, concentration of $TBAH_2PO_4$) and Method E. The program was implemented in Jupyter Notebook (v. 6.5.4) running on Python 3.11.3.

Method F

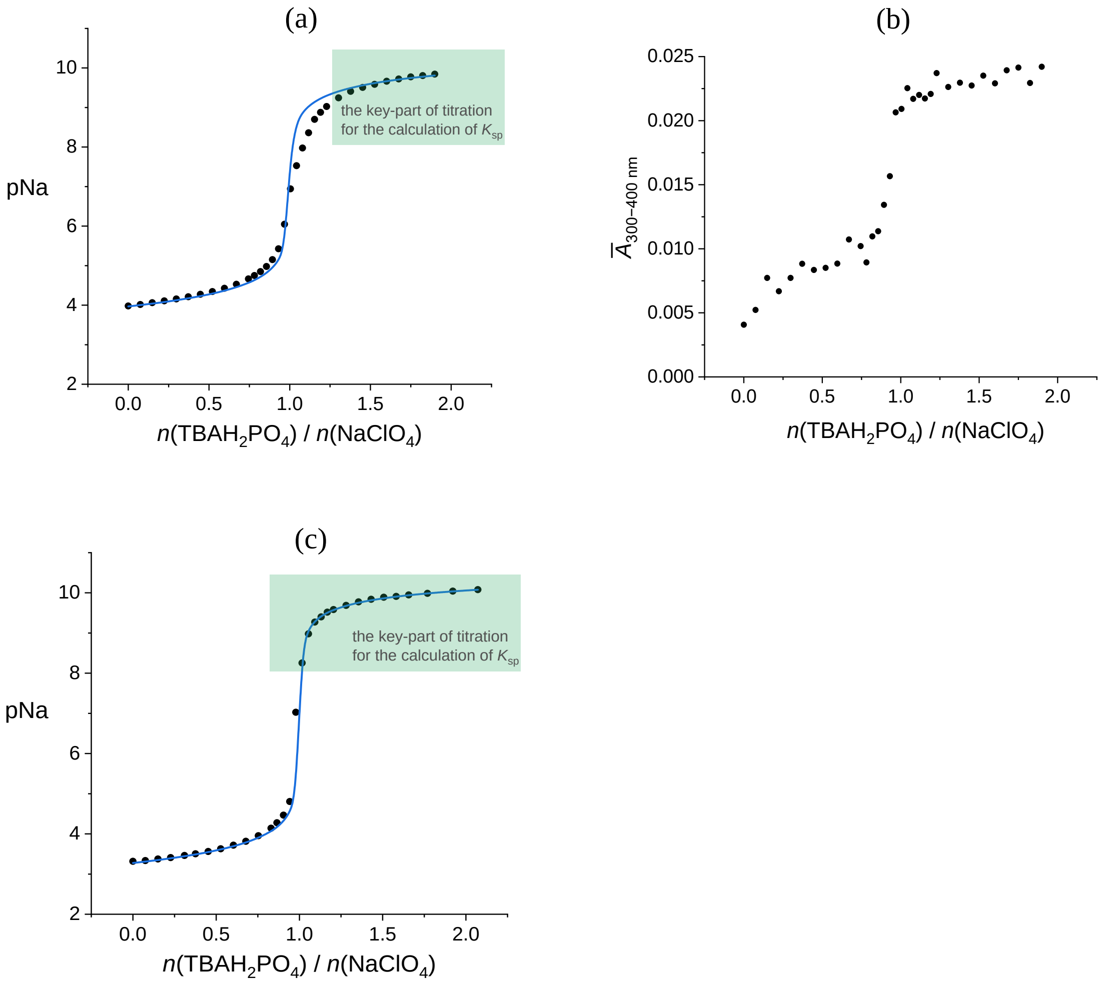

Figure S14. Potentiometric-turbidimetric titration of $NaClO_4$ with $TBAH_2PO_4$ (1.0×10^{-2} mol dm^{-3}) in acetonitrile solution of $TBAClO_4$ ($c = 1\times10^{-2}$ mol dm^{-3}, V = 25 mL). a), b) $c(NaClO_4) = 1.08\times10^{-4}$ mol dm^{-3}; c) $c(NaClO_4) = 5.31\times10^{-4}$ mol dm^{-3}. Experimental potentiometric data (●) were fitted using method F (—).

Table S2. Model used for fitting potentiometric titration data depicted in Figures 5 and S14.

reactants	product	equilibrium constant	log K
Na^+(sln) + $H_2PO_4^-$(sln)	NaH_2PO_4(sln)	K_{IP}	[7, 10][a]
$H_2PO_4^-$(sln) + $H_2PO_4^-$(sln)	$(H_2PO_4)_2^{2-}$(sln)	K_{dim}	3.38[b]
NaH_2PO_4(s)	Na^+(sln) + $H_2PO_4^-$(sln)	K_{sp}	[−14.5, −13][c]

[a] The fitting result is the same for all the values of K_{IP} in the noted interval.
[b] Literature value.[6]
[c] The interval of values through which the optimization was performed.

Physico-chemical model used in Method F

In HySS program a model was created (Table S2) using the conditions used in the specific titration (initial and final concentrations of $NaClO_4$ and $TBAH_2PO_4$). The pNa values were then calculated for single K_{sp} values ($10^{-14.5}$ – 10^{-13}, with a step of log K = 0.02) as well as the corresponding sums of squared differences between calculated and experimental pNa values (SS) for the parts of titration curves which were the most dependent on K_{sp} (shaded green in Figure S14). The value of K_{sp} was considered optimal when the corresponding SS value reached its minimum. As K_{IP} could not be evaluated solely from the potentiometric titration data, the precise value of K_{IP} was obtained in the following way. For a saturated solution of NaH_2PO_4 in pure MeCN eq. (S9) was valid and it could be rearranged to explicitly obtain K_{IP}:

$$K_{\mathrm{IP}} = (s - [\mathrm{Na}^+]_{\mathrm{sat}}) \;/\; K_{\mathrm{sp}} \qquad (S13)$$

The value of $[Na^+]_{sat}$ was determined by the following implicit function of K_{sp} and K_{dim} which was the result of a mathematical manipulation over system of equations (S5 – S10):

$$64K_{\mathrm{dim}}^2 \cdot [\mathrm{Na}^+]^6 - 256K_{\mathrm{sp}}^2K_{\mathrm{dim}}^3 \cdot [\mathrm{Na}^+]^3 - 64K_{\mathrm{sp}}^2K_{\mathrm{dim}}^2 \cdot [\mathrm{Na}^+]^2 + 256K_{\mathrm{sp}}^4K_{\mathrm{dim}}^4 = 0 \qquad (S14)$$

This polynomial equation of sixth order ($f([Na^+]) = 0$) was solved using the Excel Solver tool setting the $[Na^+]$ value as variable for optimization. To be precise, for a range of $[Na^+]$ values (10^{-12} – 10^{-9} mol dm^{-3}), $f([Na^+])$ was calculated and the $[Na^+]$ that corresponded to the value of $f([Na^+])$ closest to zero was affirmed as $[Na^+]_{sat}$ and it was further used in eq. (S13) to give the value of K_{IP}.

3. Cooperativity in binding of sodium ion pairs at host calixarene in acetonitrile

Sodium chloride

Table S3. Chemical shifts of proton signals calculated for calixarene **H** in the form of Na**H**Cl complex when fitting the ^{1}H NMR titration data depicted in Figure 7 using the approximation that all **H** is present in the form of Na**H**$^+$ at the beginning of titration and that the only existing process is complexation of Na**H**$^+$ with Cl$^–$. Comparison of the latter with the chemical shifts of the corresponding proton signals from **H** in the free form and in the forms of binary complexes Na**H**$^+$ and **H**Cl$^-$. Assignment of protons is depicted in Figure 7b.

H	**H**	Na**H**$^+$	**H**Cl$^–$	Na**H**Cl
b	8.574	7.279	9.406	9.587
a	7.020	5.392	7.344	7.074
c	7.493	7.424	/	7.499
e	6.950	7.003	6.911	6.905
n	4.620	4.686	/	4.867
hi-left	4.547	4.300	/	4.386
g	3.787	3.618	3.842	3.536

Table S4. Model used for fitting the UV spectrophotometric titration data depicted in Figure S15. The values of three complexation constants were fixed, while the fourth was refined during the fitting procedure.

reactants	product	log *K*	Fixed of refined?
Na$^+$ + **H**	Na**H**$^+$	6.69	fixed
**H** + Cl$^–$	**H**Cl$^–$	2.22	fixed
Na$^+$ + Cl$^–$	NaCl	3.41	fixed
Na$^+$ + **H +** Cl$^–$	Na**H**Cl	9.98(1)	refined

[a] Uncertainty of the last digit is given in parentheses as standard error of the mean (N = 3).

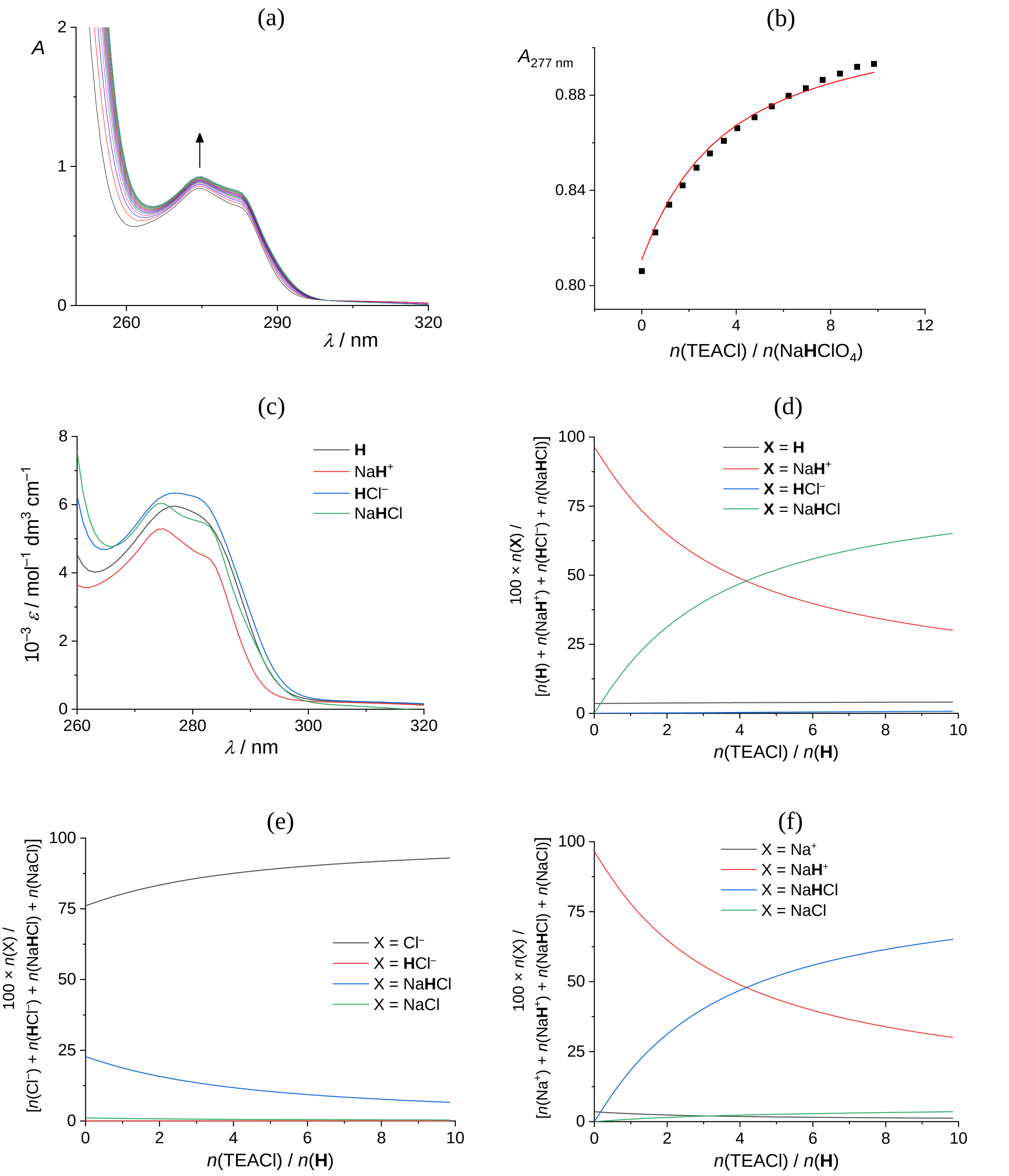

Figure S15. a) Spectrophotometric titration of Na**H**ClO4 (c(**H**) = 1.59×10^{-4} mol dm^{-3}, c(NaClO4) = 1.59×10^{-4} mol dm^{-3}, V_0 = 2.2 mL) with TEACl (c = 5.07×10^{-3} mol dm^{-3}) in acetonitrile. l = 1 cm; ϑ = (25.0 ± 0.1) °C. The spectra are corrected for dilution. b) Dependence of absorbance at 277 nm on n(TEACl) / n(Na**H**ClO4) ratio. ■ experimental; — calculated (using model given in Table S4). c) Characteristic UV spectra of **H** and its complexes with Na^+ and/or Cl^-. Distribution of species containing d) **H**, e) Cl^-, and f) Na^+ during the titration with TEACl.

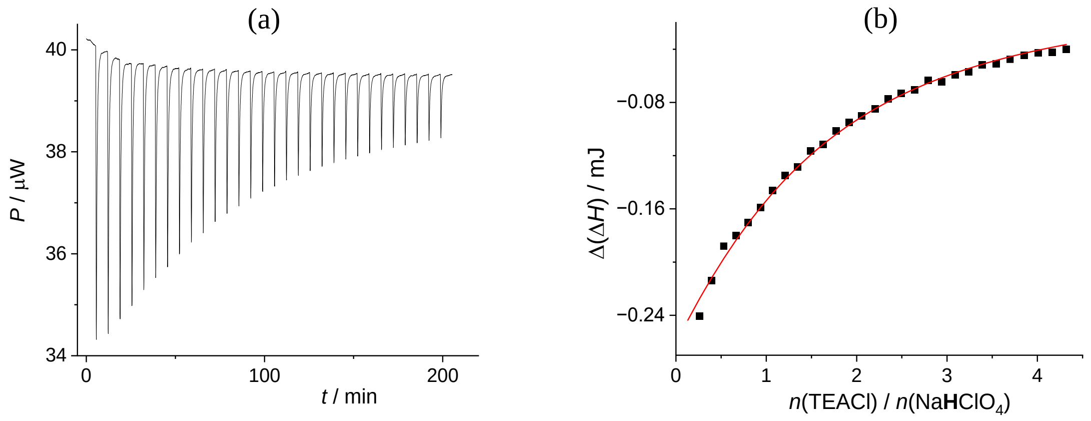

Figure S16. a) Microcalorimetric titration of Na**H**ClO4 (c(**H**) = 2.07×10^{-4} mol dm^{-3}, $c(NaClO_4)$ = 2.07×10^{-4} mol dm^{-3}, V_0 = 1.425 mL) with TEACl (c = 3.84×10^{-3} mol dm^{-3}) in acetonitrile at 25 °C; b) Dependence of successive enthalpy change on n(TEACl) / n(Na**H**ClO4) ratio. ■ experimental; — calculated. Calculation was done with the approximation that all **H** is present in the form of Na**H**$^+$ and that the only existing process is complexation of Na**H**$^+$ with Cl$^-$.

Molecular Dynamics

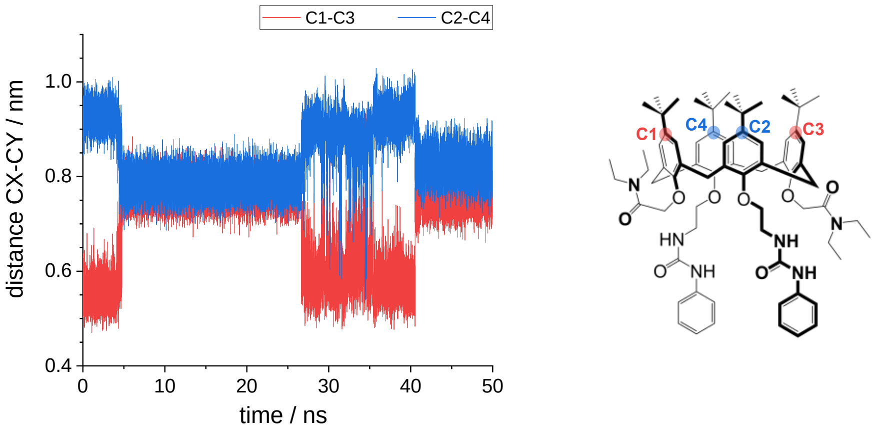

Figure S17. Distance between pairs of opposite upper rim phenyl carbons at **H** during MD simulation of free **H** (50 ns, step = 1 ps, 298.15 K, 1 bar, in MeCN).

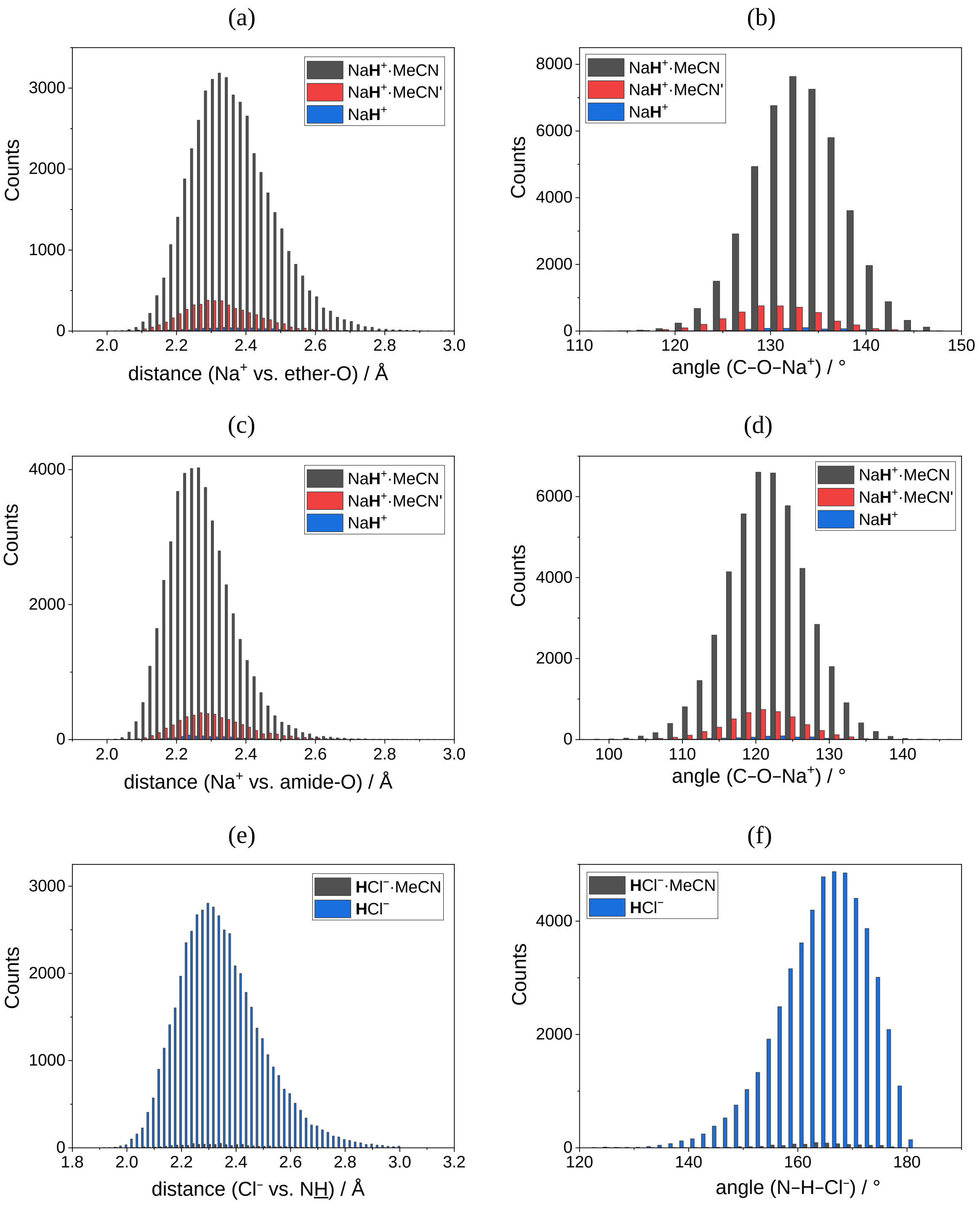

Figure S18. Histogram showing the distribution distances between Na^+ and coordinating ether (a) and amide (c) oxygens for Na**H**$^+$, and the distributions of the corresponding angles (b, d; C being the alpha-carbon). e) Histogram showing the distribution of distances between Cl^- and coordinating NH-groups for **H**Cl^-, and f) the distributions of the corresponding angles. All data were obtained by MD simulations (50 ns, step = 1 ps, 298.15 K, 1 bar, in MeCN). Histogram-bin size: 0.02 Å (a, c, e) and 2° (b, d, f). Inclusion complex written as X·MeCN refers to the case when MeCN is included in the calixarene *basket* with CH_3-end, whereas X·MeCN' indicates that the MeCN is included in the *basket* with CN-end.

Table S5. Time-averaged coordination numbers for Na^+ and Cl^-, and time-averaged numbers of intramolecular hydrogen bonds in **H**, **H**Cl^-, Na**H**$^+$, and Na**H**Cl, obtained by MD simulation (50 ns, 298.15 K, 1 bar, in MeCN).

		**H**	**H**Cl^-	Na**H**$^+$	Na**H**Cl
oxygens coordinating Na^+	total	–[a]	–[a]	6.00 ± 0.04	5.4 ± 0.9
	ether	–[a]	–[a]	4.00 ± 0.01	3.9 ± 0.3
	amide	–[a]	–[a]	2.00 ± 0.02	1.4 ± 0.8
NH groups coordinating Cl^-	total	–[a]	4.0 ± 0.1	–[a]	2.6 ± 0.9
	1st urea	–[a]	2.0 ± 0.1	–[a]	1.9 ± 0.3
	2nd urea	–[a]	2.0 ± 0.1	–[a]	0.7 ± 0.9
intramolecular hydrogen bonds	total	0.9 ± 1.0	0	0.1 ± 0.5	0.3 ± 0.6
	with amide-O	0.9 ± 1.0	0	0.1 ± 0.5	0.3 ± 0.6
	with urea-O	0.06 ± 0.28	0	0	0

[a]Not applicable. [b]Results are expressed as (average ± standard deviation).

Table S6. Structural analysis of the results of MD simulations for **H**, **H**Cl⁻, Na**H**⁺, and Na**H**Cl in acetonitrile (50 ns, 298.15 K, 1 bar).

system		**H** in MeCN		**H**Cl⁻ in MeCN		Na**H**⁺ in MeCN			Na**H**Cl in MeCN		
type of complex regarding solvent inclusion[a]		**H**·MeCN	**H**	**H**Cl⁻·MeCN	**H**Cl⁻	Na**H**⁺·MeCN	Na**H**⁺·MeCN'	Na**H**⁺	Na**H**Cl·MeCN	Na**H**Cl·MeCN'	Na**H**Cl
% simulation time		63	37	2	98	90	9	1	88	9	3
oxygens coordinating Na⁺	total	–[b]	–[b]	–[b]	–[b]	6.00 ± 0.03	6.00 ± 0.09	6.00 ± 0.04	5.3 ± 0.9	5.8 ± 0.6	4.7 ± 0.8
	ether	–[b]	–[b]	–[b]	–[b]	4.00 ± 0.01	4.00 ± 0.00	4.00 ± 0.00	3.9 ± 0.3	4.00 ± 0.02	3.9 ± 0.3
	amide	–[b]	–[b]	–[b]	–[b]	2.00 ± 0.02	2.00 ± 0.05	2.00 ± 0.04	1.4 ± 0.8	1.8 ± 0.6	0.8 ± 0.8
NH groups coordinating Cl⁻	total	–[b]	–[b]	4.0 ± 0.2	4.0 ± 0.1	–[b]	–[b]	–[b]	2.7 ± 0.9	2.2 ± 0.6	3.4 ± 0.9
	1st urea	–[b]	–[b]	2.0 ± 0.1	2.0 ± 0.1	–[b]	–[b]	–[b]	1.9 ± 0.4	2.0 ± 0.1	1.9 ± 0.3
	2nd urea	–[b]	–[b]	2.0 ± 0.1	2.0 ± 0.1	–[b]	–[b]	–[b]	0.8 ± 1.0	0.2 ± 0.6	1.5 ± 0.9
intramolecular hydrogen bonds (HB)	total	1.0 ± 0.9	0.8 ± 1.1	0	0	0.1 ± 0.5	0.05 ± 0.27	0	0.3 ± 0.7	0.01 ± 0.12	0.4 ± 0.7
	with amide-O	1.0 ± 0.9	0.7 ± 1.1	0	0	0.1 ± 0.5	0.05 ± 0.27	0	0.3 ± 0.7	0.01 ± 0.12	0.4 ± 0.7
	with urea-O	0.07 ± 0.28	0.04 ± 0.28	0	0	0	0	0	0	0	0
basket conformation[c]	$(\bar{d} \pm \sigma(d))_1$ / Å	7.6 ± 0.3	5.9 ± 0.6	7.3 ± 0.2	5.6 ± 0.3	7.9 ± 0.3	8.0 ± 0.3	7.8 ± 0.5	7.9 ± 0.3	7.9 ± 0.3	7.9 ± 0.8
	$\overline{\lvert d - d_{ref}\rvert}_1$ / Å	0.3	2.0	0.6	2.6	0.2	0.3	0.4	0.2	0.2	0.7
	$(\bar{d} \pm \sigma(d))_2$ / Å	8.0 ± 0.3	9.1 ± 0.5	8.7 ± 0.3	9.6 ± 0.3	8.0 ± 0.3	8.0 ± 0.3	7.9 ± 0.5	8.0 ± 0.3	8.0 ± 0.3	7.6 ± 0.8
	$\overline{\lvert d - d_{ref}\rvert}_2$ / Å	0.3	1.2	0.9	1.7	0.3	0.3	0.4	0.3	0.3	0.7
number of exchanged MeCN molecules		2	–[b]	1	–[b]	19	13	–[b]	13	9	–[b]
structure with intramolecular HB(s) / % total simulation time		36	15	0	0	5.6	0.6	0	16.6	0.1	0.7
		51		0		6.2			17.4		

representative clusters<br>% total simulation time / coordination pattern[d] / type of complex regarding solvent inclusion	**H** in MeCN			**H**Cl⁻ in MeCN			Na**H**⁺ in MeCN			Na**H**Cl in MeCN		
	27	HB (0,0,0)	**H**·MeCN	97	Cl (4,2,2)<br>HB (0,0,0)	**H**Cl⁻	84	Na (6,4,2)<br>HB (0,0,0)	Na**H**⁺·MeCN	48	Na (6,4,2)<br>Cl (2,2,0)<br>HB (0,0,0)	Na**H**Cl·MeCN
	25	HB (2,2,0)	**H**·MeCN				9	Na (6,4,2)<br>HB (0,0,0)	Na**H**⁺·MeCN'	13	Na (4,4,0)<br>Cl (4,2,2)<br>HB (0,0,0)	Na**H**Cl·MeCN
	23	HB (0,0,0)	**H**							8	Na (6,4,2)<br>Cl (2,2,0)<br>HB (0,0,0)	Na**H**Cl·MeCN'

[a] Inclusion complex written as X·MeCN refers to the case when MeCN is included into the calixarene *basket* with CH_3-end, whereas X·MeCN' indicates that the MeCN is included into the *basket* with CN-end. [b] Not applicable. [c] With $\bar{d}$ is denoted the average distance between opposing aryl carbon atoms connected to the *tert*-butyl groups, σ(*d*) represents the standard deviation of *d*, and d_{ref} = 7.85 Å corresponds to C_{4v} *cone* conformation.[7] [d] Coordination patterns: oxygens coordinating Na⁺ = Na (total, ether, amide), NH groups coordinating Cl⁻ = Cl (total, 1st urea, 2nd urea), intramolecular hydrogen bonds = HB (total, with amide-O, with urea-O). The full form of coordination patterns used for clustering is represented in Table S7.

Table S7. Representative clusters of structures obtained by MD simulation (50 ns, 298.15 K, 1 bar, in MeCN) for **H**, **H**Cl⁻, Na**H**⁺, and Na**H**Cl. Coordination patterns describing each cluster are denoted in abbreviated and full form, with the latter being used in the clustering algorithm. Description of full coordination patterns: Na (total-O, ether-O, amide-O, urea-O), Cl (total-NH, urea-1, urea-2), HB (total-NH, total with amide-O, urea-1 with amide-O1, urea-2 with amide-O1, urea-1 with amide-O2, urea-2 with amide-O2, total with urea-O, urea-1 with urea-O2, urea-2 with urea-O1).

	% total simulation time	type of complex regarding solvent inclusion	coordination pattern	
			abbrevated	full
**H**	27	**H**·MeCN	HB (0,0,0)	HB (0,0,0,0,0,0,0,0,0)
	25	**H**·MeCN	HB (2,2,0)	HB (2,2,0,2,0,0,0,0,0)
	23	**H**	HB (0,0,0)	HB (0,0,0,0,0,0,0,0,0)
**H**Cl⁻	97	**H**Cl⁻	Cl (4,2,2)<br>HB (0,0,0)	Cl (4,2,2)<br>HB (0,0,0,0,0,0,0,0,0)
Na**H**⁺	84	Na**H**⁺·MeCN	Na (6,4,2)<br>HB (0,0,0)	Na (6,4,2,0)<br>HB (0,0,0,0,0,0,0,0,0)
	9	Na**H**⁺·MeCN'	Na (6,4,2)<br>HB (0,0,0)	Na (6,4,2,0)<br>HB (0,0,0,0,0,0,0,0,0)
Na**H**Cl	49	Na**H**Cl·MeCN	Na (6,4,2)<br>Cl (2,2,0)<br>HB (0,0,0)	Na (6,4,2,0)<br>Cl (2,2,0)<br>HB (0,0,0,0,0,0,0,0,0)
	13	Na**H**Cl·MeCN	Na (4,4,0)<br>Cl (4,2,2)<br>HB (0,0,0)	Na (4,4,0,0)<br>Cl (4,2,2)<br>HB (0,0,0,0,0,0,0,0,0)
	8	Na**H**Cl·MeCN'	Na (6,4,2)<br>Cl (2,2,0)<br>HB (0,0,0)	Na (6,4,2,0)<br>Cl (2,2,0)<br>HB (0,0,0,0,0,0,0,0,0)

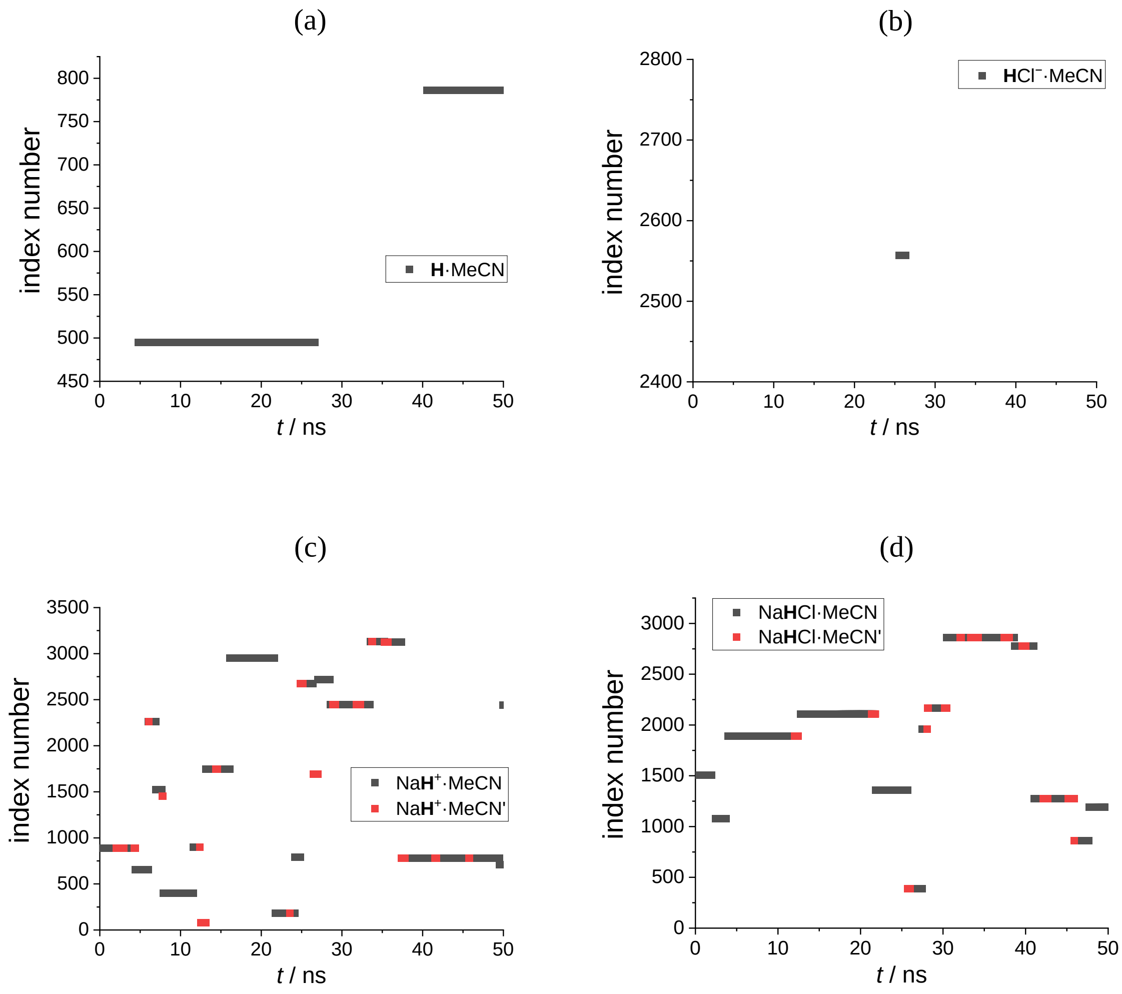

Figure S19. Index number of acetonitrile molecules that occupy the hydrophobic cavity of **H** during MD simulations of a) **H**, b) **H**Cl^-, c) Na**H**$^+$, and d) Na**H**Cl in acetonitrile (50 ns, 298.15 K, 1 bar).

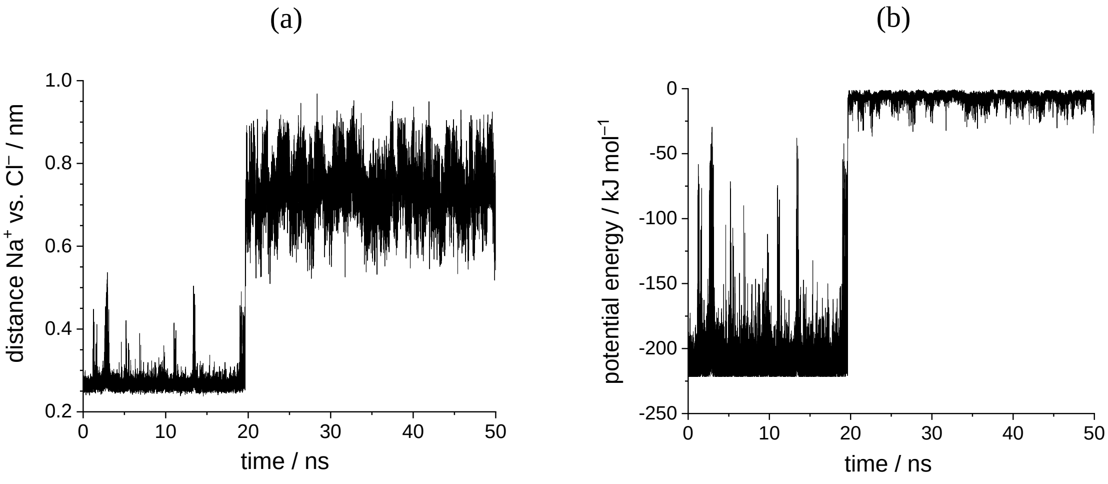

Figure S20. a) Distance and b) potential energy between Na^{+} and Cl^{-} at **H** during MD simulation of Na**H**Cl (50 ns, step = 1 ps, 298.15 K, 1 bar, in MeCN). Potential energy consists of four terms: Coulomb -SR and -14, Lennard Jones -SR and -14, all calculated using Gromacs.

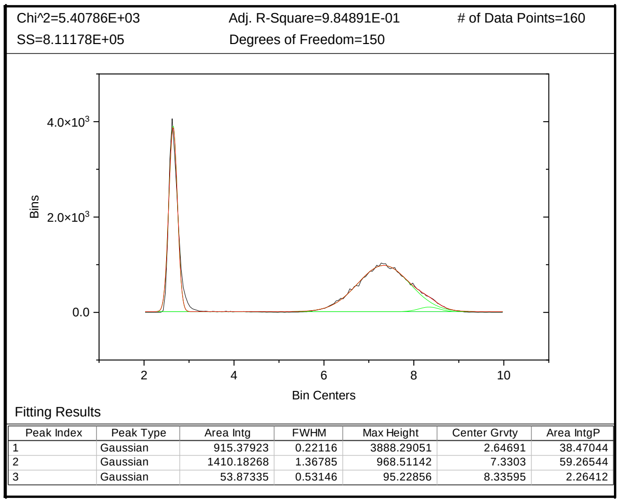

Peak Index	Peak Type	Area Intg	FWHM	Max Height	Center Grvty	Area IntgP
1	Gaussian	915.37923	0.22116	3888.29051	2.64691	38.47044
2	Gaussian	1410.18268	1.36785	968.51142	7.3303	59.26544
3	Gaussian	53.87335	0.53146	95.22856	8.33595	2.26412

Figure S21. The distribution analysis results for the distances between Na^{+} and Cl^{-} (Figure 14a in the main text of this study) obtained by MD simulations (50 ns, step = 1 ps, 298.15 K, 1 bar, in MeCN). Analysis was conducted using the Peak Analyzer algorithm in the OriginPro software.

(a) (b)

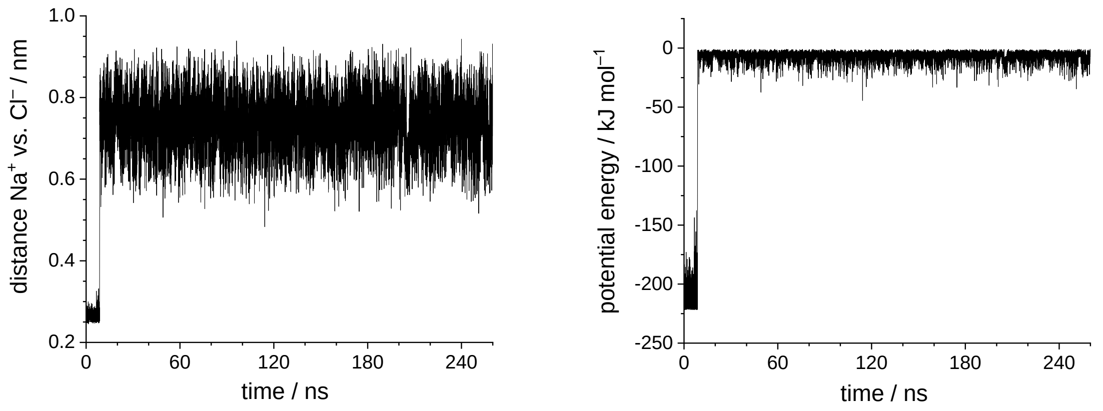

Figure S22. a) Distance and b) potential energy between Na^+ and Cl^- at **H** during MD simulation of Na**H**Cl (260 ns, step = 10 ps, 298.15 K, 1 bar, in MeCN). Potential energy consists of four terms: Coulomb -SR and -14, Lennard Jones -SR and -14, all calculated using Gromacs.

(a) (b)

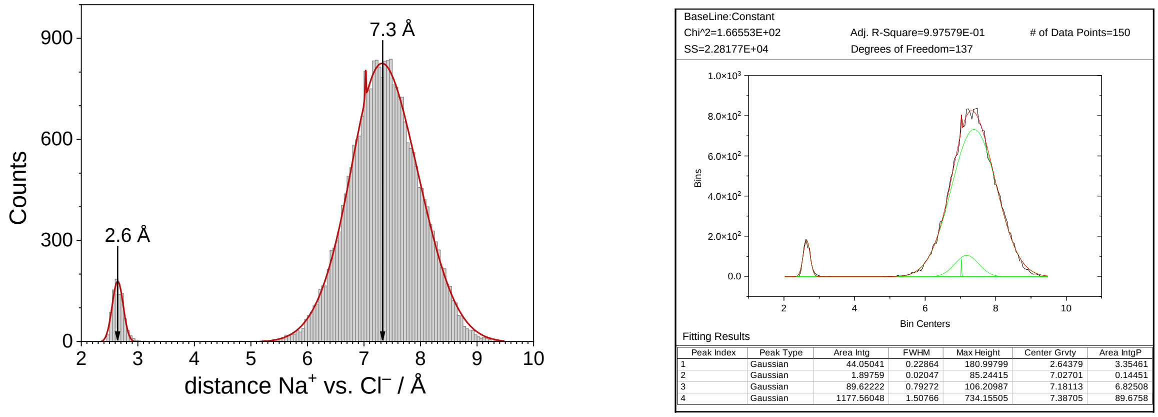

Peak Index	Peak Type	Area Intg	FWHM	Max Height	Center Grvty	Area IntgP
1	Gaussian	44.05041	0.22864	180.99799	2.64379	3.35461
2	Gaussian	1.89759	0.02047	85.24415	7.02701	0.14451
3	Gaussian	89.62222	0.79272	106.20987	7.18113	6.82508
4	Gaussian	1177.56048	1.50766	734.15505	7.38705	89.6758

Figure S23. a) Histogram showing the distribution of distances between Na^+ and Cl^- obtained by MD simulations (260 ns, step = 10 ps, 298.15 K, 1 bar, in MeCN). Histogram-bin size: 0.05 Å. b) Analysis was conducted using the Peak Analyzer algorithm in the OriginPro software.

Sodium hydrogen sulfate

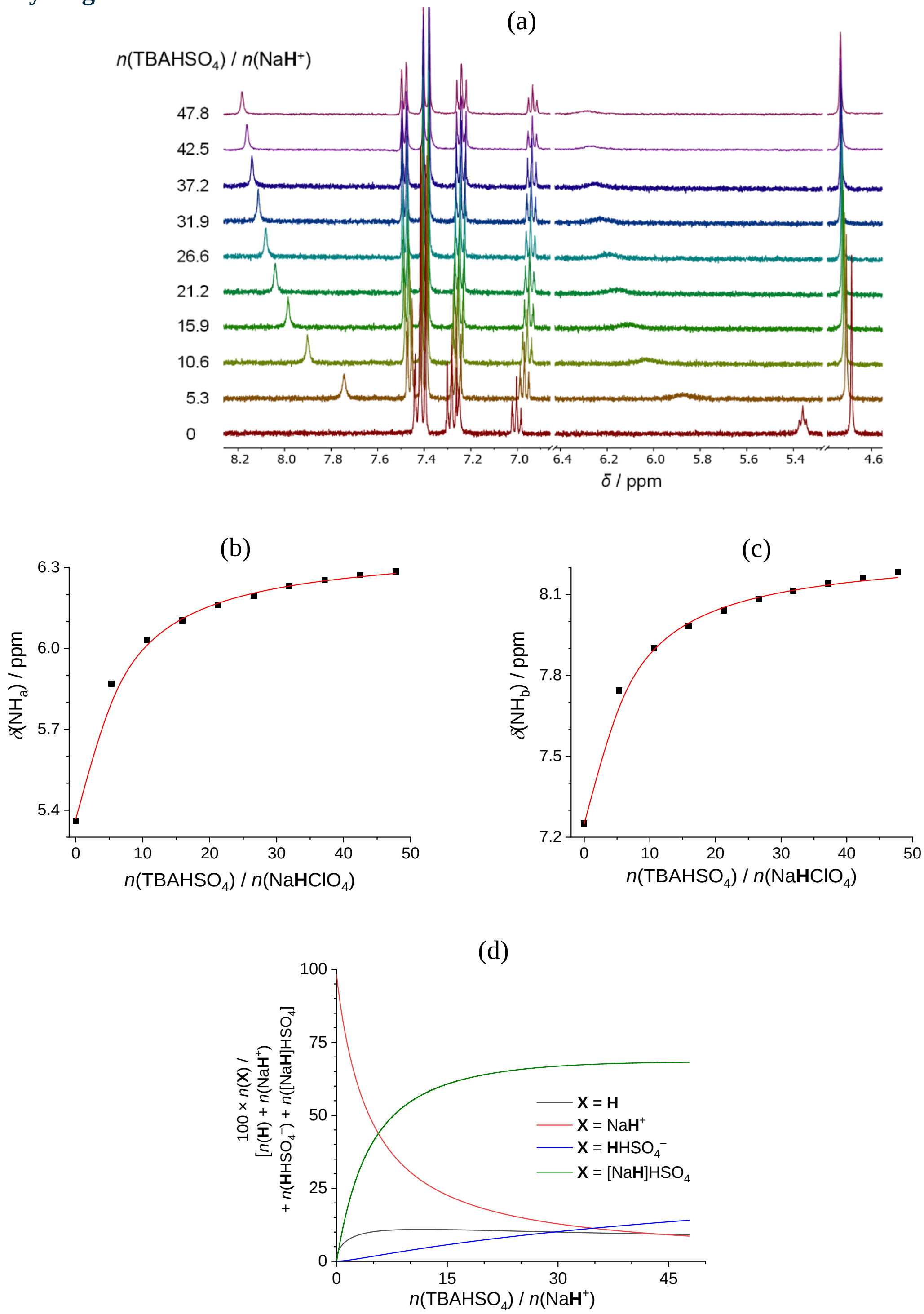

Figure S24. a) ^{1}H NMR spectroscopy titration of Na**H**ClO_4 (c = 6.51×10^{-4} mol dm^{-3}, V_0 = 531 µL) with $TBAHSO_4$ (c = 0.368 mol dm^{-3}) in CD_3CN at 25 °C. b), c) Experimental (■) and calculated (—) chemical shifts. Calculation was done with the approximation that all **H** is present in the form of Na**H**$^+$ and that the only existing process is complexation of Na**H**$^+$ with HSO_4^-. d) Distribution of **H** and its complexes with Na^+ and/or HSO_4^- during the titration with $TBAHSO_4$ (calculated with HySS program) using more complex model demonstrated in Table S9 with log β(**H**HSO_4^-) = 1.74[8] and log β(Na**H**HSO_4) = 9.14.

Table S8. Chemical shifts of proton signals calculated for calixarene **H** in the form of Na**H**HSO_4 complex when fitting the ^{1}H NMR titration data depicted in Figure S24 using the approximation that all **H** is present in the form of Na**H**$^+$ at the beginning of titration and that the only existing process is complexation of Na**H**$^+$ with Cl^-. Comparison of the latter with the chemical shifts of the corresponding proton signals from **H** in the free form and in the forms of binary complexes Na**H**$^+$ and **H**HSO_4^-. Assignment of protons is depicted in Figure 7b.

H	**H**	Na**H**$^+$	**H**HSO_4^-	Na**H**HSO_4
b	8.574	7.279	/	8.279
a	7.020	5.392	/	6.394
c	7.493	7.424	8.573	7.504
e	6.950	7.003	6.775	6.929
n	4.620	4.686	/	4.738
hi-left	4.547	4.300	/	4.345

Table S9. Model used for fitting the UV spectrophotometric titration data depicted in Figure S26. The values of two complexation constants were fixed, while the other two were refined during the fitting procedure.

reactants	product	log β	Fixed of refined?
Na^+ + **H**	Na**H**$^+$	6.69	fixed
**H** + HSO_4^-	**H**HSO_4^-	1.72	fixed
Na^+ + HSO_4^-	$NaHSO_4$	4.3(1)	refined
Na^+ + **H** + HSO_4^-	Na**H**HSO_4	9.09(1)	refined

[a] Uncertainty of the last digit is given in parentheses as standard error of the mean (N = 3).

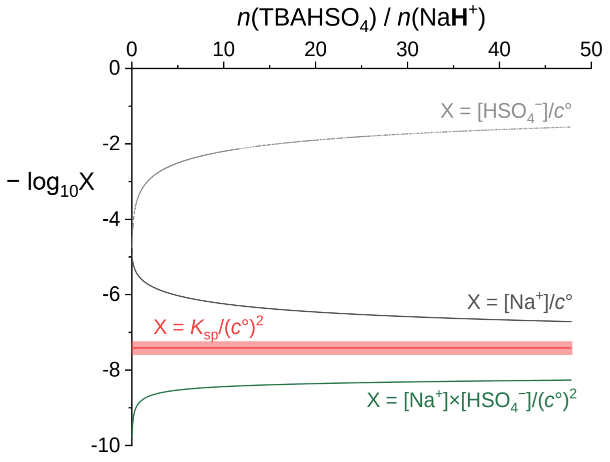

Figure S25. Simulation of ^{1}H NMR titration of Na**H**ClO_4 with $TBAHSO_4$ depicted in Figure S24 using complex model demonstrated in Table S9. Results of simulation show that the product of concentrations of free Na^+ and HSO_4^- is below K_{sp}.

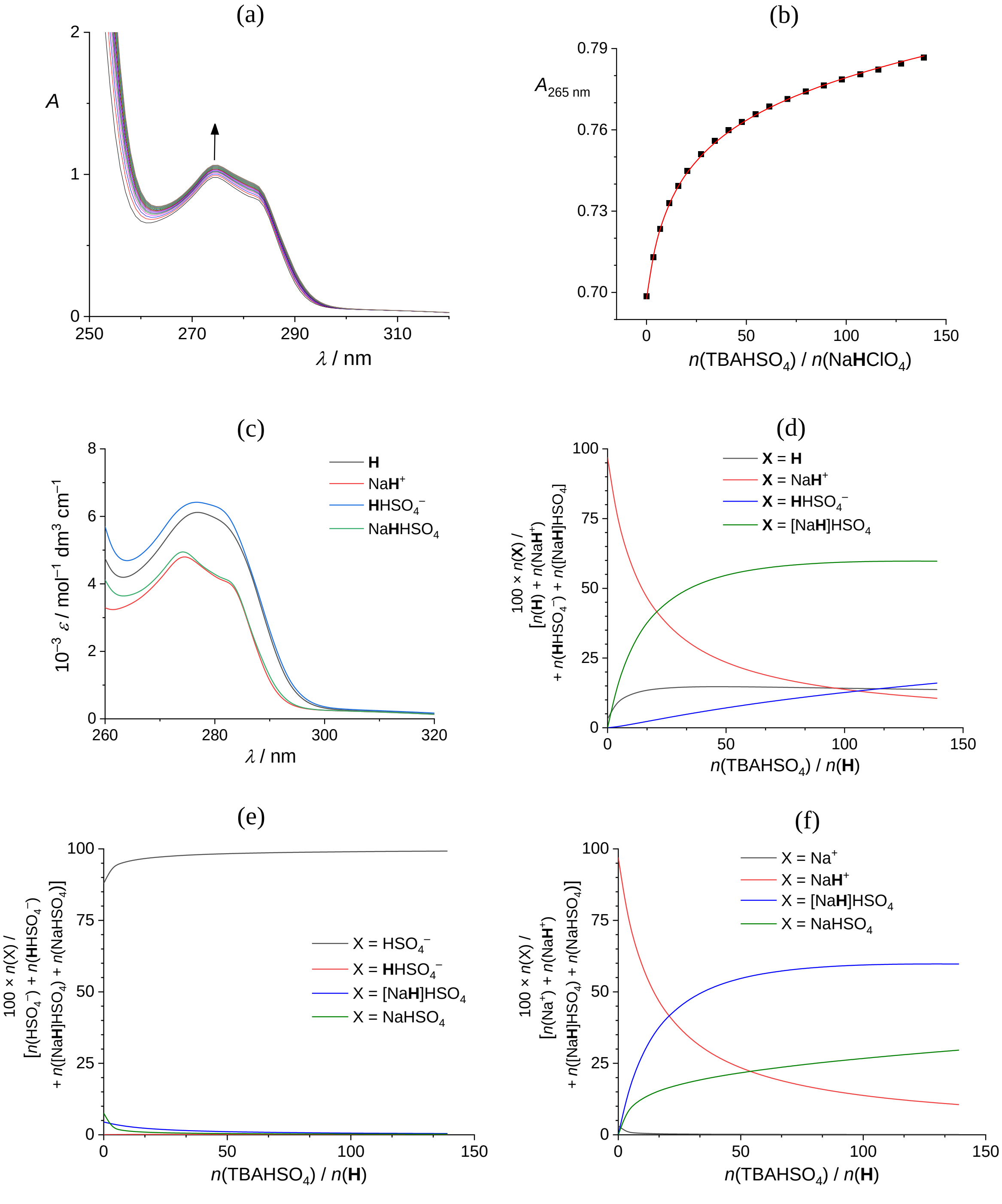

Figure S26. a) Spectrophotometric titration of Na**H**ClO4 ($c = 2.02 \times 10^{-4}$ mol dm^{-3}, $V_0 = 2.2$ mL) with TBAHSO4 ($c = 0.101$ mol dm^{-3}) in acetonitrile. $l = 1$ cm; $\vartheta = (25.0 \pm 0.1)$ °C. The spectra are corrected for dilution. b) Dependence of absorbance at 265 nm on n(TBAHSO4) / n(Na**H**ClO4) ratio. ■ experimental; — calculated (using complete model presented in Table S9). c) Characteristic UV spectra of **H** and its complexes with Na^+ and/or HSO_4^-. Distribution of species containing d) **H**, e) HSO_4^-, and f) Na^+ during the titration with TBAHSO4.

Sodium dihydrogen phosphate

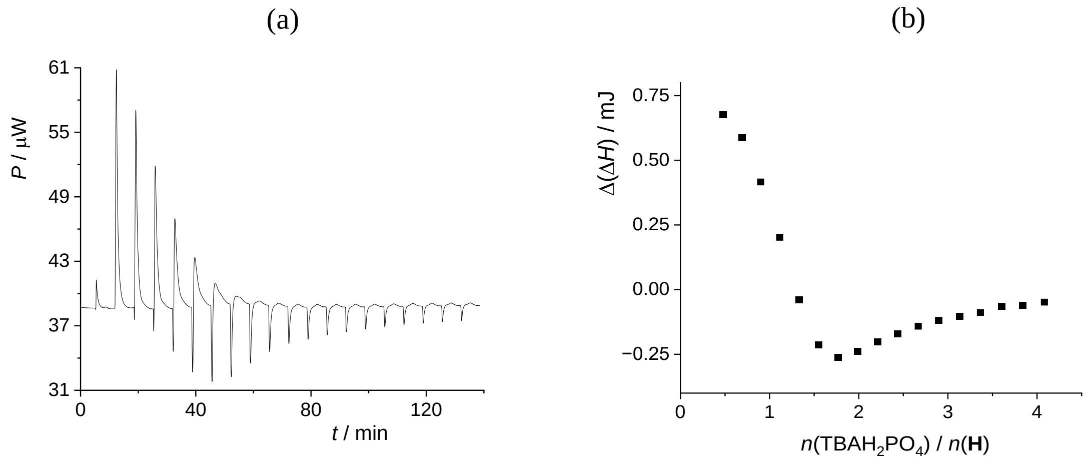

Figure S27. Microcalorimetric titration of Na**H**ClO_4 (c = 1.95×10^{-4} mol dm^{-3}, V_0 = 1.432 mL) with TBAH_2PO_4 (c = 3.78×10^{-3} mol dm^{-3}) in acetonitrile at 25 °C; b) Dependence of successive enthalpy change on n(TBAH_2PO_4) / n(**H**) ratio. This was a failed trial of quantification of cooperativity in the binding of $H_2PO_4^-$ at Na**H**$^+$ vs. **H**. The failing-factor was the precipitation of NaH_2PO_4 (endothermic signals) which was the dominant process due to the very low value of K_{sp} and the high value of K_{IP} (both independently evaluated).

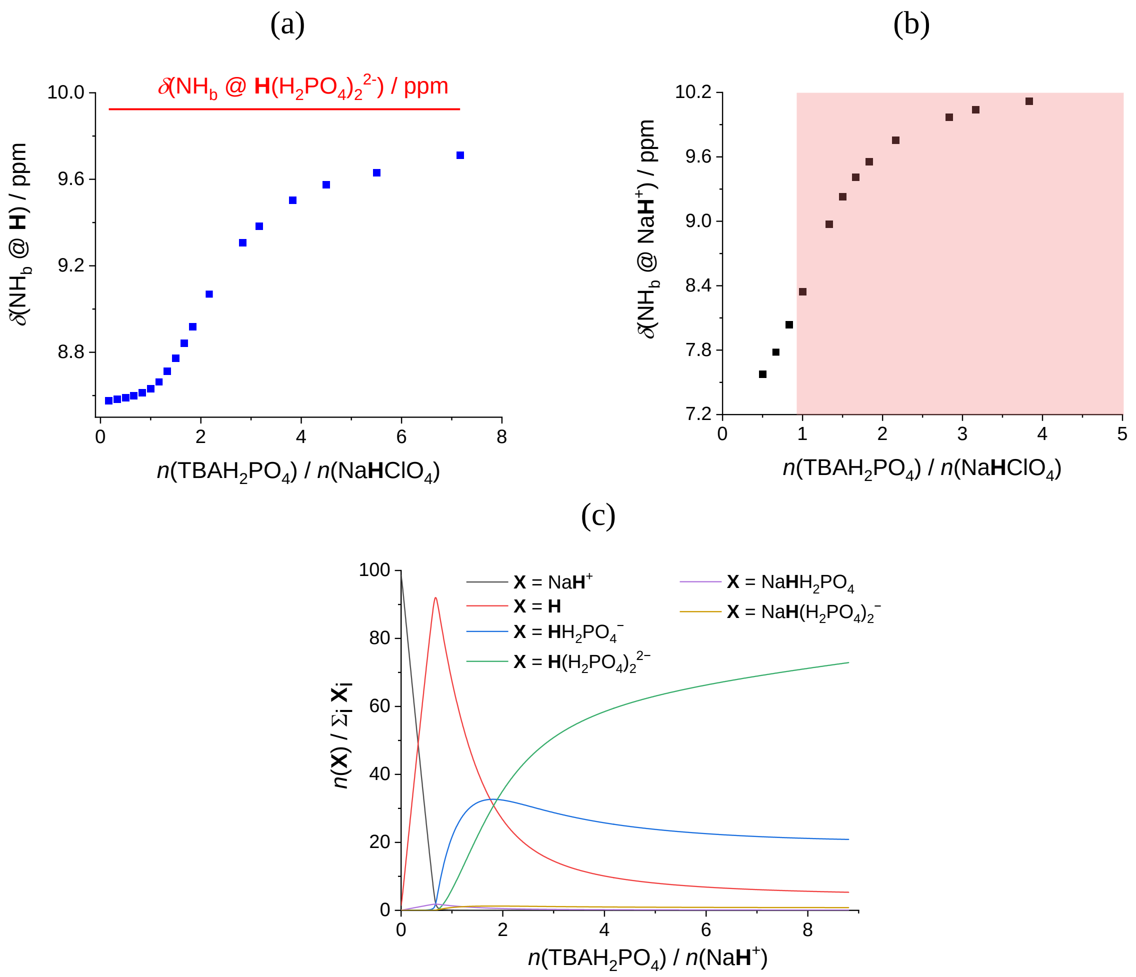

Figure S28. ^{1}H NMR spectroscopy titration of Na**H**ClO4 (c = 8.97×10^{-4} mol dm^{-3}, V_0 = 490 μL) with TBAH2PO4 (c = 1.46×10^{-2} mol dm^{-3}) in CD3CN at 25 °C. a), b) Chemical shifts of ureido protons at **H** and Na**H**$^+$. c) Distribution of species containing **H** during the titration in the case of quite high cooperativity in binding of Cl^- at Na**H**$^+$ (model is written in Table S10). It is clear that not even cooperativity can explain the long existence of Na**H**$^+$ signals (according to speciation diagram, signals in red area of titration curve b) should not exist). A possible cause of this experimental fact could be slow kinetics in precipitation of NaH_2PO_4. However, no quantification of complexation behavior of Na**H**$^+$ towards $H_2PO_4^-$ was possible.

Table S10. Model used for creating distribution depicted in Figure S28d.

reactants	product	log β
Na^+(sln) + $H_2PO_4^-$(sln)	NaH_2PO_4(sln)	8.1
Na^+(sln) + **H**(sln)	Na**H**$^+$(sln)	6.69
$H_2PO_4^-$(sln) + $H_2PO_4^-$(sln)	$(H_2PO_4)_2^{2-}$(sln)	3.38
**H**(sln) + $H_2PO_4^-$(sln)	**H**$H_2PO_4^-$(sln)	3.71
**H**(sln) + 2$H_2PO_4^-$(sln)	**H**$(H_2PO_4)_2^{2-}$(sln)	7.37
Na**H**$^+$(sln) + $H_2PO_4^-$(sln)	Na**H**H_2PO_4(sln)	12
Na**H**$^+$(sln) + 2$H_2PO_4^-$(sln)	Na**H**$(H_2PO_4)_2^-$(sln)	16

4. Literature

[1] K. Leko, A. Usenik, N. Cindro, M. Modrušan, J. Požar, G. Horvat, V. Stilinović, T. Hrenar, V. Tomišić, *ACS Omega* **2023**, *8*, 43074–43087.

[2] G. Horvat, V. Stilinović, T. Hrenar, B. Kaitner, L. Frkanec, V. Tomišić, *Inorg. Chem.* **2012**, *51*, 6264–6278.

[3] J. Požar, I. Nikšić-Franjić, M. Cvetnić, K. Leko, N. Cindro, K. Pičuljan, I. Borilović, L. Frkanec, V. Tomišić, *J. Phys. Chem. B* **2017**, *121*, 8539–8550.

[4] J. Požar, M. Cvetnić, A. Usenik, N. Cindro, G. Horvat, K. Leko, M. Modrušan, V. Tomišić, *Molecules* **2022**, *27*, 470.

[5] K. Izutsu, *Electrochemistry in Nonaqueous Solutions*, Wiley-VCH Verlag GmbH & Co. KGaA, Weinheim, **2002**.

[6] N. Bregović, N. Cindro, L. Frkanec, K. Užarević, V. Tomišić, *Chem. – Eur. J.* **2014**, *20*, 15863–15871.

[7] G. Horvat, L. Frkanec, N. Cindro, V. Tomišić, *Phys. Chem. Chem. Phys.* **2017**, *19*, 24316–24329.

[8] M. Cvetnić, N. Cindro, N. Bregović, V. Tomišić, *ACS Phys. Chem. Au* **2024**, *4*, 773–786.
